# Grape polyphenols reduce fasting glucose and increase hyocholic acid in healthy humans: a meta-omics study

**DOI:** 10.1038/s41538-025-00443-6

**Published:** 2025-05-27

**Authors:** Esther Mezhibovsky, Guojun Wu, Yue Wu, Zhibin Ning, Karen Bacalia, Sriya Sadangi, Riddhi Patel, Alexander Poulev, Rocio M. Duran, Marie Macor, Susette Coyle, Yan Y. Lam, Ilya Raskin, Daniel Figeys, Liping Zhao, Diana E. Roopchand

**Affiliations:** 1 Department of Food Science and New Jersey Institute for Food, Nutrition and Health (Rutgers Center for Lipid Research), School of Environmental and Biological Sciences, Rutgers, The State University of New Jersey, New Brunswick, NJ 08901 USA; 2 Department of Biochemistry and Microbiology and New Jersey Institute for Food, Nutrition, and Health, School of Environmental and Biological Sciences, Rutgers, The State University of New Jersey, New Brunswick, NJ 08901 USA; 3 Ottawa Institute of Systems Biology and Department of Biochemistry, Microbiology and Immunology, Faculty of Medicine, University of Ottawa, Ottawa, ON Canada; 4 Department of Plant Biology, School of Environmental and Biological Sciences, School of Environmental and Biological Sciences, Rutgers University, New Brunswick, NJ 08901 USA; 5 Department of Surgery, Rutgers Robert Wood Johnson Medical School (RWJMS), New Brunswick, 08903 NJ USA; 6 Department of Medicine, School of Clinical Medicine, LKS Faculty of Medicine, The University of Hong Kong, Hong Kong SAR, China

**Keywords:** Mass spectrometry, Proteomics, Metagenomics, Microbial ecology, Microbiome

## Abstract

Grape polyphenols (GPs) are rich in B-type proanthocyanidins, which promote metabolic resilience. Longitudinal metabolomic, metagenomic, and metaproteomic changes were measured in 27 healthy subjects supplemented with soy protein isolate (SPI, 40 g per day) for 5 days followed by GPs complexed to SPI (GP-SPI standardized to 5% GPs, 40 g per day) for 10 days. Fecal, urine, and/or fasting blood samples were collected before supplementation (day –5), after 5 days of SPI (day 0), and after 2, 4 and 10 days of GP-SPI. Most multi-omic changes observed after 2 and/or 4 days of GP-SPI intake were temporary, returning to pre-supplementation profiles by day 10. Shotgun metagenomics sequencing provided insights that could not be captured with 16S rRNA amplicon sequencing. Notably, 10 days of GP-SPI decreased fasting blood glucose and increased serum hyocholic acid (HCA), a glucoregulatory bile acid, which negatively correlated with one gut bacterial guild. In conclusion, GP-induced suppression of a bacterial guild may lead to higher HCA and lower fasting blood glucose.

## Introduction

Dietary polyphenols may promote metabolic health through multiple mechanisms, including their ability to modulate host-digestive enzyme activity^1,2^, promote antioxidant levels^3,4^, and alter the gut microbial community^5,6^. B-type proanthocyanidin (PAC) compounds are a major class of grape polyphenols (GPs) found in Concord grape berries (*Vitis labrusca*) and pomace, which consist of the skins and seeds that are discarded after juice production. Epicatechin and catechin monomers (i.e., flavan-3-ols) linked by single interflavan bonds lead to the formation of B-type PACs, also called condensed tannins, that range in size from dimers (e.g. PACB1—PACB8 having same mass but different molecular configurations)^7^ to oligomers (trimers and tetramers) and polymers^8^. PACs have been categorized as anti-nutrients, based on reports that they can reduce metabolism and/or bioavailability of carbohydrate, proteins, and minerals such as iron, copper and zinc, in particular when they are isolated from the food matrix^9,10^. However, PACs consumed within a food matrix are less likely to lead to nutrient or mineral deficiency and demonstrate a range of health benefits^9,11^. Despite having low bioavailability, preclinical studies indicate that GPs can positively impact metabolic health by altering the gut microbial community^5,12^, bile acids (BAs)^12^, and the intestinal mucus layer^13^. Bacterial catabolism of diverse polyphenols yields a smaller group of polyphenol-derived microbial metabolites, mainly phenolic acids with increased bioavailability^14–16^ and bioactivity^17–19^. Gut bacteria metabolize mainly PAC dimers and flavan-3-ol monomers while PACs with higher degrees of polymerization are largely resistant to bacterial catabolism^20^.

**Fig. 1:**
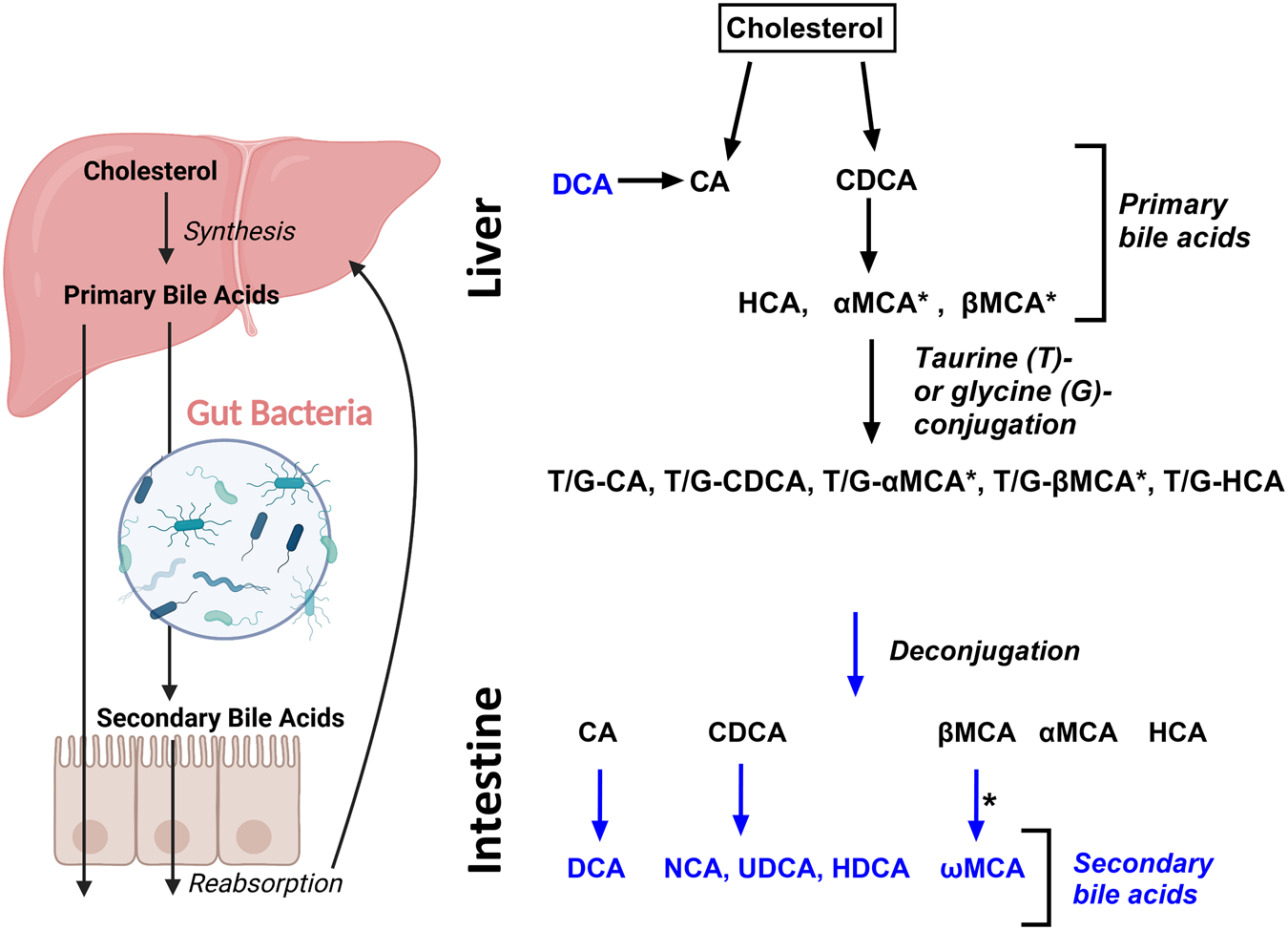
Bile acid conversions. Primary bile acids, including cholic acid (CA), chenodeoxycholic acid (CDCA), hyocholic acid (HCA), alpha-muricholic acid (αMCA), and beta-muricholic acid (βMCA), are synthesized in the liver from cholesterol and conjugated with taurine (T) or glycine (G). BAs are released from the liver postprandially to facilitate nutrient absorption and can also be metabolized by gut bacteria into secondary bile acids including deoxycholic acid (DCA), nutricholic acid (NCA), ursodeoxycholic acid (UDCA), hyodeoxycholic acid (HDCA), omega-muricholic acid (ωMCA) [left]. Conversions between bile acid species [right]. Asterisks are placed next to conversions known to occur in rodents, but synthesis is uncertain in humans. Arrows indicate involvement of intermediate reactions which may occur via one or more steps.

Primary BAs (PBAs) synthesized in hepatocytes from cholesterol are released postprandially into the gut to aid in absorption of lipophilic compounds. While 95% of BAs are reabsorbed in the ileum, ~5% are metabolized by colonic bacteria to secondary BAs (SBAs, Fig. 1). PBAs and SBAs have emerged as important signaling molecules with varied hydrophilicity, ligand functions, and diverse effects on nutrient metabolism^21,22^. The best characterized BA receptors are nuclear transcription factor farnesoid X receptor (FXR) and Takeda G protein-coupled receptor 5 (TGR5), which both have roles in regulating glucose metabolism^21,22^.

Prior studies have demonstrated that dietary polyphenols from a variety of sources can be extracted and sorbed to a protein food matrix, such as soy protein isolate (SPI), allowing standardized delivery of polyphenols with increased stability, bioaccessibility, and bioavailability compared to polyphenol extract alone^23–27^. Compared to control mice fed high-fat diet (HFD) formulated with SPI, mice fed an isocaloric HFD formulated with GPs complexed to SPI (GP-SPI) exhibited greater resistance to weight gain, adiposity, and glucose intolerance in association with increased relative abundance of *Akkermansia muciniphila*^5^. Directly supplementing mice or humans with *A. muciniphila* was shown to improve metabolic outcomes^28,29^. Compared to *db/db* mice fed low-fat diet (LFD) formulated with SPI, mice fed LFD supplemented with GP-SPI showed improved oral glucose tolerance in association with increased relative abundance of *A. muciniphila*, decreased levels of serum SBAs, and reduced expression of FXR responsive genes that regulate BA synthesis and glucose metabolism^12^. LFD-fed mice that received daily oral administration of GPs extracted from Concord grape pomace or purified grape seed PACs (each delivering 360 mg PAC/kg/day) showed increased relative abundance of fecal and cecal *A. muciniphila* within 10 days, indicating that B-type PAC compounds were sufficient to induce this microbiome change^6^.

Dietary PAC intake is highly variable across different populations and geographical regions. PAC intake in the general US population was estimated to be 54–95 mg/day^30,31^ with higher intakes (257 mg/day) for young to middle-aged US women^32^. In contrast, PAC intake in the Spanish population was estimated at 450 mg/day^33^, reflecting differences in American vs. Mediterranean dietary patterns. Few clinical trials have been performed to investigate the specific effects of PAC-rich extracts on the gut microbiota and microbial metabolites^34–36^. Here we tested the hypothesis that 10 days of GP-SPI consumption would increase the relative abundance of *A. muciniphila* in the human gut microbiota. Furthermore, we aimed to develop a biological signature of GP-SPI supplementation in humans and therefore investigated whether GPs would induce broader changes to the metabolome, gut microbiome, and metaproteome profiles that could contribute to metabolic health.

## Results

### Participant demographics and effect of GP-SPI on CMP values, stool, and food intake patterns

**Fig. 2:**
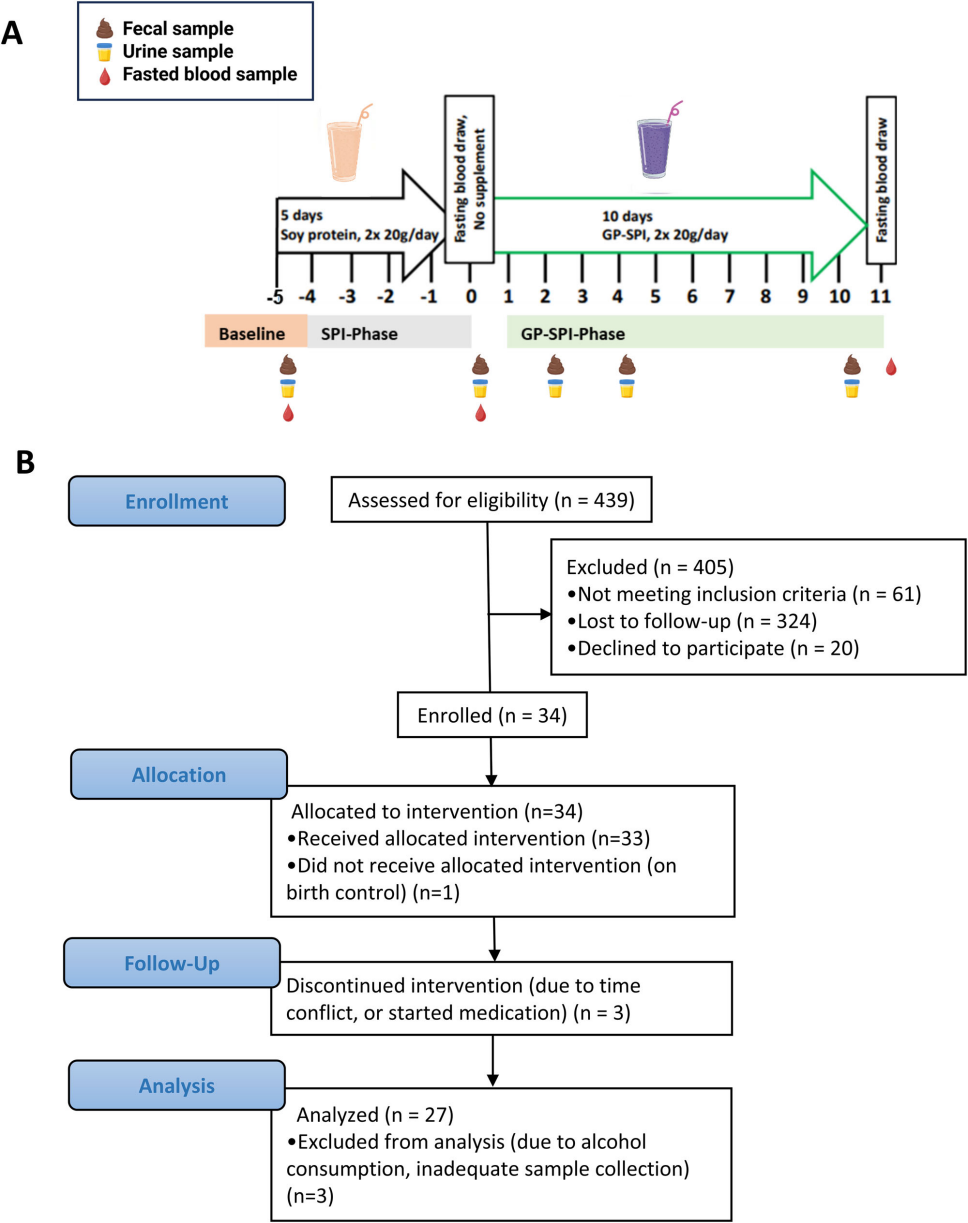
Study design. **A** This was a single arm longitudinal study designed to identify biological signatures of GP supplementation using a multi-omics approach. Participants were supplemented with SPI (day –5 to day –1) followed by a break from supplementation (day 0) and then supplementation with GP-SPI (day 1 to day 10). Fecal, urine, or blood samples were collected at the indicated time points. **B** Flow diagram reporting enrollment, allocation, follow-up and analysis of participants involved in this single-arm longitudinal study.

This single-arm, 17-day longitudinal time course study (Fig. 2A) was carried out in accordance with the Declaration of Helsinki and was approved by the Rutgers University eIRB (Pro2018002579) and registered with ClinicalTrials.gov (NCT04018066). Recruitment began in June and ended in December 2019. The flow chart shows the numbers of subjects that were screened and enrolled (Fig. 2B). After screening, consent, and a fasting comprehensive metabolic panel (CMP) test to confirm general health, 34 subjects were enrolled and 30 subjects completed the study, but samples from 3 subjects were excluded from sample analyses due to protocol non-compliance (Fig. 2A). Data were therefore analyzed from 27 individuals, i.e., 11 males and 16 females. Of the subjects, 55.6% were Asian, 18.51% were White, 11.11% were Black or African American, 3.7% identified as more than one race, and 11.11% reported race unknown, or not reported. Participants had a mean BMI of 23 ± 3.1 kg/m^2^ and mean age of 22 ± 4 years (Table 1).

**Table 1:** Participant characteristics

	# of individuals
**Ethnicity**	
Hispanic or Latino	4
Not Hispanic or Latino	22
Unknown or unreported	1
**Race**	
Asian	15
Black or African American	3
White	5
More than one race	1
Unknown or not reported	3
**Sex**	
Female	16
Male	11
**Age (years)**	
18	5
19	2
20	4
21	7
22	1
23	1
24	2
25	1
26	2
29	1
32	1
**BMI**	
18	2
19	4
20	3
21	4
22	2
23	0
24	3
25	1
26	5
27	2
28	1

*BMI* body mass index.

There were no adverse effects from GP-SPI consumption based on self-reports or CMP results obtained from fasting blood samples collected on morning of day 11, after 10 days of GP-SPI supplementation (Table 2). Compared to pre-intervention baseline values, all CMP values remained within normal ranges after GP-SPI supplementation although fasting glucose, potassium, and chloride were significantly decreased, and Blood Urea Nitrogen (BUN) and anion gap were significantly increased (Table 2). Supplementation did not alter participant stool characteristics (Supplemental Fig. 1A-C), as determined by the Bristol stool scale questionnaire.

**Table 2:** Comprehensive Metabolic Panel (CMP) results before supplementation (baseline) and after GP-SPI supplementation

Parameter	Normal range	Baseline Mean ± SD	Day 11 Mean ± SD	*p*-value	Sig.
Glucose	70–100 mg/dL	88.78 ± 6.247	85.96 ± 6.791	0.0453	*
BUN	6–23 mg/dL	12.81 ± 4.297	14.81 ± 3.282	0.0114	*
Creatine	0.5–1.2 mg/dL	0.7593 ± 0.1907	0.7593 ± 0.1845	>0.9999	ns
Calcium	8.6–10.4 mg/dL	9.819 ± 0.3783	9.641 ± 0.5308	0.1004	ns
Sodium	136–145 mmol/L	142.7 ± 2.284	142.2 ± 2.326	0.3762	ns
Potassium	3.5–5.0 mmol/L	4.826 ± 0.3381	4.574 ± 0.4266	0.0144	*
Chloride	98–108 mmol/L	103.4 ± 2.803	102.3 ± 1.710	0.0443	*
Total CO2	22–30 mmol/L	26.04 ± 1.720	25.51 ± 1.694	0.0890	ns
Anion Gap	7–17 mEq/L	13.26 ± 2.194	14.41 ± 1.907	0.0116	*
Protein	6–8 g/dL	7.381 ± 0.3669	7.374 ± 0.3265	0.9075	ns
Albumin	3.5–5.5 g/dL	4.826 ± 0.3358	4.870 ± 0.2959	0.4654	ns
Bilirubin Total	0.1–1.2 mg/dL	0.5370 ± 0.2589	0.5370 ± 0.2498	>0.9999	ns
Alkaline phosphatase (ALP)	45–115 IU/L	68.56 ± 15.19	70.70 ± 16.79	0.1696	ns
Aspartate transaminase (AST)	10–55 IU/L	21.26 ± 5.404	21.41 ± 5.597	0.9073	ns
Alkaline transaminase (ALT)	10–50 IU/L	16.89 ± 7.439	17.52 ± 8.591	0.5907	ns

Statistical significance was determined by a paired t-test for normally distributed data, or by a Wilcoxon test for nonparametric data, based on Shapiro-Wilk’s test for normal distribution.

*p < 0.05.

*ns* not significant.

Participants abstained from PAC-rich foods during the study and kept daily digital diaries to track all foods and drinks they consumed. A total of 24 complete food intake data sets were collected from participants and this information was entered into the Food Processor Analysis Software to estimate the average caloric intake of each participant. Total caloric intake did not differ by day, or between the periods of SPI and GP-SPI supplementation (Supplementary Fig. 2A, B). Based on Euclidean distance between subject’s dietary patterns throughout the study, three groups could be distinguished by their intake of grains, vegetables, fruits, dairy, and protein (Supplementary Fig. 2C–F). Group 1 consumed less protein, dairy, vegetables, and fruit than groups 2 and 3. Group 3 consumed more dairy than groups 1 and 2. Grain intake was similar across all groups.

### PAC-derived microbial metabolites detected after GP-SPI supplementation

**Fig. 3:**
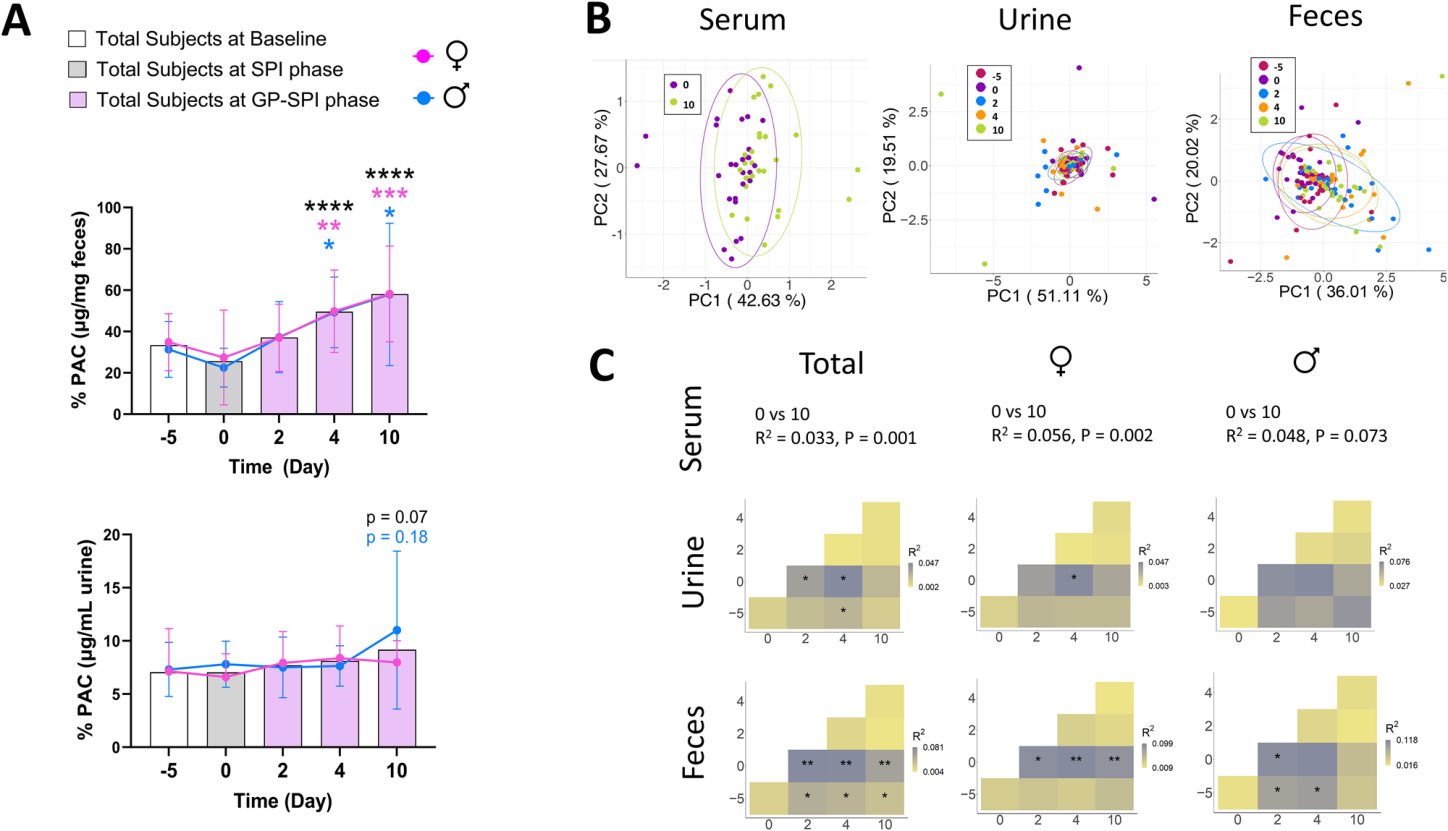
PAC-derived bacterial metabolites detected in serum, urine, and fecal samples. **A** PACB2 as a percentage of total polyphenols measured in urine and fecal samples. **B** adjusted PCoA (aPCoA) plot of Euclidean distance between PAC-derived microbial metabolites detected by LC-MS in serum, urine, and feces. **C** Subject stratified PERMANOVA test results for metabolite profiles in serum, urine, and feces of total participant pool, females, or males.

Compared to day –5, 0, or 2, the PAC levels in feces were significantly increased on days 4 and 10 while PAC levels in urine remained constant across all days suggesting no increase in absorption (Fig. 3A, bottom row). Increased fecal PACs on days 4 and 10 confirmed that participants consumed GP-SPI and complied with study protocol.

Supplementary Fig. 3 illustrates a subset of previously reported gut bacteria-derived metabolites of B-type PAC dimers^20,37–44^. To confirm that GPs dissociated from the SPI food matrix and were available for gut bacterial catabolism, we quantified 5-(3′,4′-dihydroxyphenyl)-γ-valerolactone (DHPV), a major metabolite of PAC dimers^40^, and six additional polyphenol-derived metabolites that may be derived from PACs, as well as other classes of polyphenols (Supplementary Fig. 4). aPCoA plots, corrected for inter-individual variations, revealed that differences in PAC/polyphenol-derived metabolite profiles across time points could be detected in serum and feces, but not urine (Fig. 3B). Subject-stratified PERMANOVA testing revealed that, compared to day 0, serum levels of PAC/polyphenol-derived metabolites were significantly different after 10 days of GP-SPI supplementation in all participants and in females, with a trending difference in males (Fig. 3C). Subject-stratified PERMANOVA testing showed that compared to day 0, the metabolite profile in urine was altered only on days 2 and 4 with changes driven mainly by female participants (Fig. 3C). Compared to day 0, the metabolite profile in feces was altered on days 2, 4, and 10, with changes driven mainly by female participants for days 4 and 10 (Fig. 3C).

The seven quantified PAC/polyphenol-derived microbial metabolites were detected in fecal samples after GP-SPI intake resulting in increased fecal levels of DHPV, 3-phenylpropionic acid (3-PPA), para-coumaric acid (*p*CA), vanillic acid (VA), desaminotyrosine (DAT), and 3-hydroxyphenylacetic acid (3-HPA), but not 4-hydroxybenzoic acid (4-HBA) (Supplementary Fig. 4). Unchanged 4-HBA levels over the GP-SPI phase suggests it was produced from dietary polyphenols other than PACs^45^ (Supplementary Fig. 4). Detection of all metabolites at baseline (day –5) and after the 5-day SPI supplementation phase (while subjects abstained from PAC-rich foods) indicated that diverse classes of dietary polyphenols may be biotransformed to similar metabolite compounds, consistent with prior reports^45^. Fecal levels of DAT increased only at day 2 and were not significantly changed in serum or urine (Supplementary Fig. 4).

Serum levels of DHPV and 3-PPA were increased, suggesting intestinal absorption and delivery to circulation (Supplementary Fig. 4). GP-SPI intake resulted in increased urine concentrations of DHPV, but not 3-PPA, suggesting DHPV may have accelerated urinary excretion compared to 3-PPA (Supplementary Fig. 4). During GP-SPI supplementation, 3-HPA and *p*CA levels were increased in urine, but not serum (Supplementary Fig. 4), suggesting urinary excretion of these compounds was greater compared to DHPV and 3-PPA, which remained elevated in circulation. After 10 days of GP-SPI intake, fecal levels of VA was increased, but trended lower in serum (Supplementary Fig. 4). Females had approximately twice the concentration of 3-HPA in urine compared to males at day 10 (mean of 68.34 vs. 34.42 µg/mL, *p* < 0.05; Supplementary Table 1) while males had about twofold more VA in feces compared to females at day 2 (0.022 vs. 0.0095 µg/mg feces, *p* < 0.05; Supplementary Table S2), and it remains to be determined if differences are due to sexual dimorphism or individual differences in the production and/or absorption of these microbial metabolites.

Unique patterns in production, absorption, and excretion of microbial metabolites were observed across individuals. Nine participants (i.e., 1, 3, 7, 10, 13, 15, 17, 19, and 25) had no DHPV in serum at day 0 or 10, though this metabolite was detected in their fecal and urine samples. Despite having evidence of DAT production in feces, eight participants (i.e., participants 3, 6, 8, 15, 17, 19, 25, and 26), had no DAT levels detected in serum at day 0 or 10, and nine subjects (i.e., participants 2, 3, 5, 7, 13, 15, 19, 25, and 26), had no detectable DAT in urine at any timepoint. One subject (participant 10) had no detectable DAT in feces or serum at any timepoint, but curiously did have detectable levels in urine. Four participants (i.e., 17, 21, 23 and 26) had no detectable 3-HPA in feces at any timepoint but had this metabolite in other compartments. Only participant 16 had no detectable *p*CA in urine at any timepoint. These data indicate that heterogeneous human microbiomes produce varied metabolite profiles which may be further influenced by host genetic polymorphisms affecting metabolite absorption^46^.

### GP-SPI supplementation modulated serum BA profiles

To gain insight into how GP-SPI supplementation would alter BAs in serum, a panel of 25 BAs were quantified using pure standards. 18 of 25 BAs were detected in serum (Supplementary Table 3), suggesting that the 7 BAs not detected in serum (i.e., taurocholic acid (TCA), tauro-alpha-muricholic acid (TαMCA), tauro-beta-muricholic acid (TβMCA), lithocholic acid (LCA), taurohyodeoxycholic acid (THDCA), tauro-omega-muricholic acid (TωMCA), tauro-ursodeoxycholic acid (TUDCA)) remained exclusively in enterohepatic circulation, or were present below the limit of detection.

**Fig. 4:**
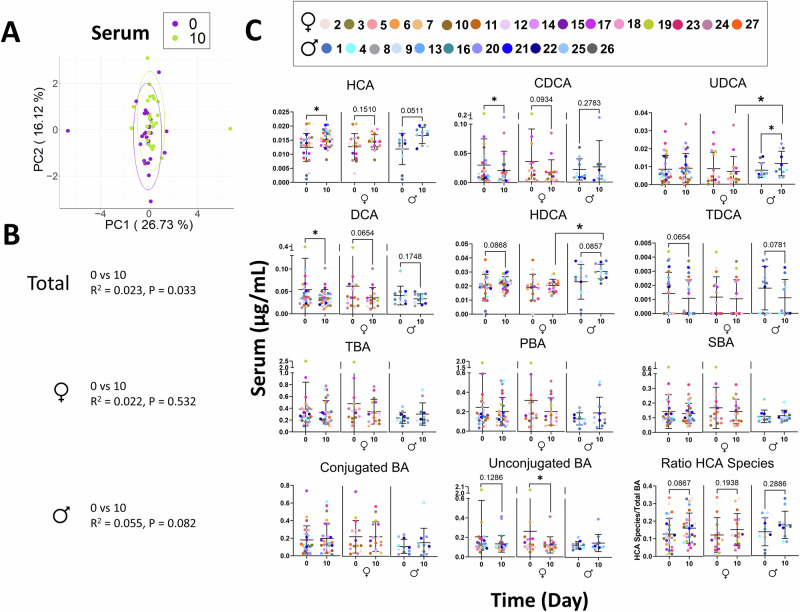
SPI and GP-SPI supplementation altered serum and fecal bile acid (BA) profiles. **A** Adjusted PCoA (aPCoA) plots of serum BAs at day 0 and 10. **B** Subject stratified PERMANOVA test results based on Euclidean distance of BA profile in serum of total participant pool, females, or male groups. **C** Individual BAs that were significantly changed in serum of total participants pool (left panel), females (middle panel), and males (right panel). Total BA (TBA) included LCA, HCA, HDCA, UCDCA, CDCA, DCA, GUDCA, GCDCA, GDCA, GLCA, TUDCA, TCDCA, TLCA, ωMCA, αMCA, βMCA, CA, GHCA, GCA, THDCA, TωMCA, TβMCA, and TCA. BA were also summed as primary BAs (PBA), secondary BAs (SBA), conjugated BA, or unconjugated BA and the ratio of total HCA species detected from total BA. Significance was determined by Wilcoxon test for nonparametric data or paired *t* test for parametric data, based on normality of data as determined by Shapiro-Wilks test.

When serum BA profiles were adjusted for inter-individual variability, analysis of the aPCoA plots revealed that serum BA profiles of the participants were distinguishable across timepoints (Fig. 4A). Subject stratified PERMANOVA testing performed on all participants showed that GP-SPI intake significantly changed the serum BA profile (day 0 vs. day 10, *p* = 0.033, Fig. 4B). When data were analyzed according to sex, 10 days of GP-SPI intake did not alter the serum BA profile in females but there was a trending difference in males (*p* = 0.082, Fig. 4B).

Individual BA changes were analyzed in serum for all participants and for males and females separately. Independent of SPI or GP-SPI supplementation, sex-specific BA changes were detected at one to three timepoints for serum levels of ursodeoxycholic acid (UDCA) and hyodeoxycholic acid (HDCA) (Supplementary Table 4). After 10 days of GP-SPI intake, serum HCA was significantly increased in all participants (*p* < 0.05), a change that occurred mainly in males (*p* = 0.0537), compared to females (*p* = 0.159; Fig. 4C). Prior studies have reported that HCA species (i.e., HCA, HDCA, GHCA, THCA, GHDCA, THDCA) comprise 2–3% of total serum BAs in humans but 76% of total serum BAs in pigs, which are highly resistant to metabolic disease^47^. Levels of HCA and other HCA species were found to be inversely related to fasting glucose levels in humans, mice, and pigs leading to the suggestion that HCA may be a useful biomarker to assess risk of metabolic disease^47,48^. 10 days of GP-SPI supplementation resulted in a trending increase (*p* = 0.0867; Fig. 4C) in the proportion of serum HCA species (i.e., HCA, HDCA, and GHCA) relative to total BAs.

GP-SPI supplementation increased serum levels of UDCA in males but not females (Fig. 4C). GP-SPI supplementation decreased the levels of CDCA in all participants although significance was lost when males and females were separated (Fig. 4C). GP-SPI supplementation decreased serum levels of DCA in all subjects, but the significance was strongest in females (Fig. 4C). GP-SPI supplementation did not alter serum TBA, PBA, SBA, or conjugated BAs; unconjugated serum BAs were reduced in females only (Fig. 4C). The relationship between PAC consumption, changes in BA species, and fasting glucose warrants further investigation in a larger cohort of participants.

### GP-SPI supplementation had mainly transient effects on gut bacterial communities

Shot-gun metagenomic sequencing was performed on fecal samples collected before any supplementation (day –5), after 5 days of SPI intake (day 0), and after 2, 4, or 10 days of GP-SPI intake (*n* = 27 samples per timepoint). To characterize the strain-level/sub-species level changes in the gut microbiota, 1433 high-quality draft genomes were de novo assembled for the metagenomic dataset. After integrating the genomes from Human Gastrointestinal Bacteria Genome Collection (HGG)^49^ to improve the metagenomic analysis, 1638 non-redundant high-quality draft metagenomic assembled genomes (HQMAGs) were obtained. After read recruitment for abundance estimation, 1635 HQMAGs with abundance information were kept for further analysis.

### Genome-based analyses

**Fig. 5:**
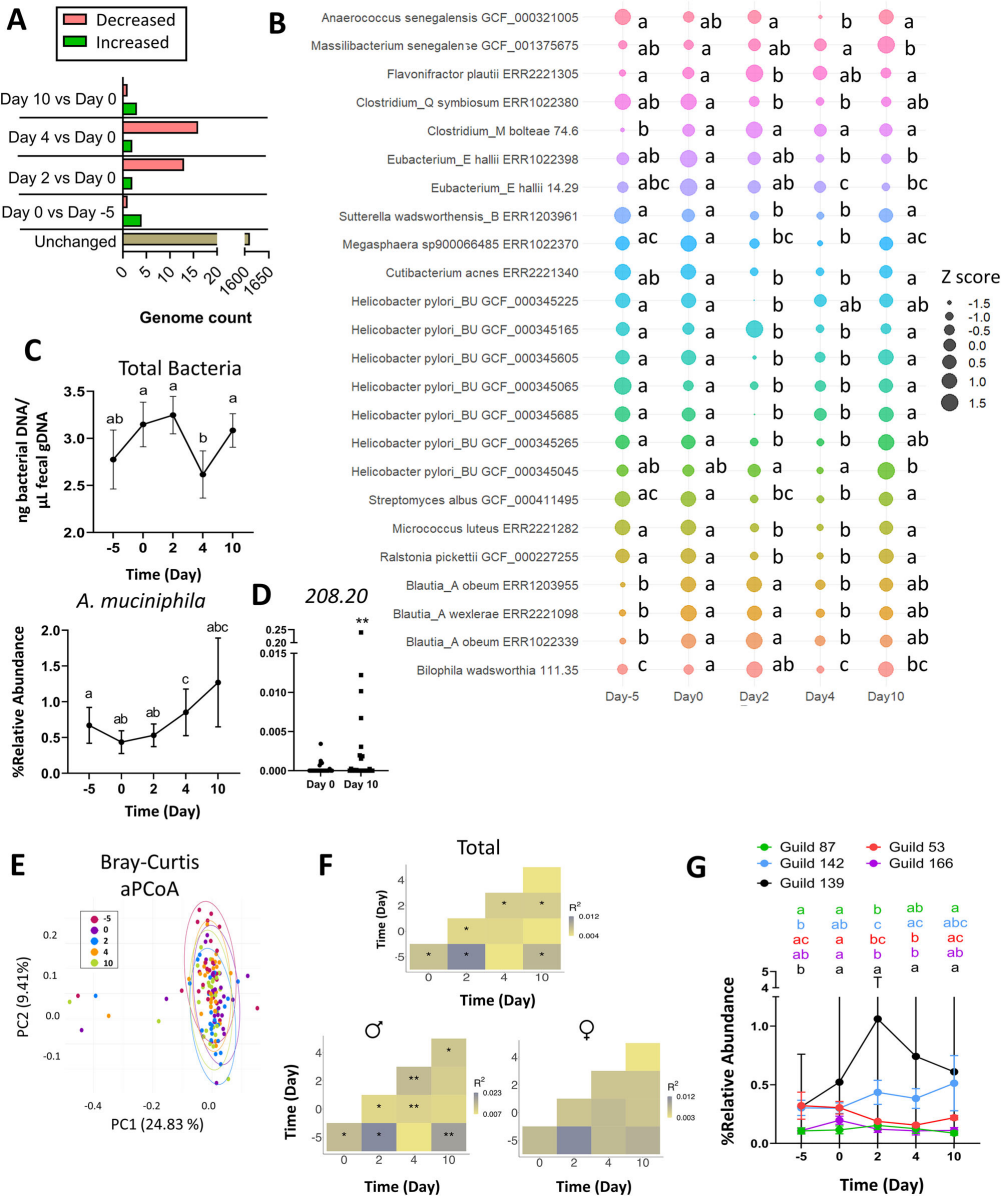
Effects of SPI and GP-SPI on fecal gut bacterial genomes and guilds. Fecal microbial guilds and *Akkermansia muciniphila* were altered by SPI or GP-SPI supplementation. **A** Number of up- or down-regulated fecal microbial genomes detected by shotgun sequencing after SPI supplementation (Day 0 vs. Day –5) or after GP-SPI supplementation (Day 0 vs. Day 2, 4, or 10), based on a threshold of q ≤ 0.25. **B** Bubble-plot of bacterial strains that were significantly changed by GP-SPI supplementation as determined by screening with Benjamini Hochberg test, q ≤ 0.25. Friedman test followed by Nemenyi post hoc test was then applied to detect significant difference between GP-SPI phase timepoints and day 0 (*p* < 0.05). **C** qPCR quantification of total bacteria (top) and relative abundance of *A. muciniphila* species (bottom) in fecal samples collected at indicated time points. Data are mean ± SD. Different letters denote statistical significance (*p* < 0.05) determined by Nemenyi post hoc test following Friedman test. **D** Relative abundance of *A. muciniphila 208.20*, as detected by shot*-*gun sequencing, at day 0 and 10, with statistical significance (**p < 0.005) determined by a Wilcoxon *t* test. **E** Adjusted PCoA (aPCoA) plot of bacterial guilds based on Bray Curtis dissimilarity. **F** Subject stratified PERMANOVA test results based on Bray-Curtis dissimilarity for total participant pool, males only, or females only. **G** Change in relative abundance (%) of bacterial guilds (mean ± SEM) by GP-SPI, as detected by shotgun sequencing. Data are mean ± SD. One-way ANOVA followed by the Benjamini Hochberg post-hoc test was used to screen for a significant effect of SPI or GP-SPI (q ≤ 0.25). Friedman test followed by Nemenyi’s post hoc test was used to detect guilds significantly altered by SPI (day –5 vs. 0) or GP-SPI-supplementation (Day 0 vs. Day 2, 4, or 10).

Alpha diversity was not altered by SPI supplementation (day –5 vs. day 0) nor by GP-SPI supplementation (day 0 vs. days 2, 4, and 10, Supplementary Fig. 5A). Genome-level beta diversity, expressed as PCoA plots based on Bray-Curtis (differentiating by genome presence and abundance; Supplementary Fig. 5B) or Jaccard (differentiating by genome presence only; Supplementary Fig. 5C) dissimilarity metrics, showed that samples collected from the same subject clustered together rather than clustering by supplementation or time of sample collection. The clustering of samples from the same individual is consistent with previous reports that gut microbiomes from different persons, including identical twins, are unique^50^. Significant genome-level difference between timepoints across the 27 participants was more visible after adjustment for sources of inter-individual variation in aPCoA plots and was confirmed by subjected-stratified PERMANOVA tests (Supplementary Fig. 5D, E, Supplementary Table 5). From the 1635 genomes, abundance of only 24 genomes (<2%) was differentially altered across the study (Benjamini Hochberg’s test, q ≤ 0.25), being either transiently suppressed or increased by GP-SPI intake (Fig. 5A, see Supplementary Table 6 for identified HQMAGs). GP supplementation suppressed the levels of several bacterial strains on days 2 and 4, but most significant reductions were lost by day 10 as abundances returned to day 0 levels (Fig. 5A).

Most bacterial genomes were unaffected after 5 days of SPI supplementation (day –5 vs day 0), although there were significant increases in abundances of three *Blautia* strains and in *Clostridium bolteae 74.6*, and decreased abundance of *Bilophila wadsworthia 111.35* strain (Fig. 5B; Supplementary Table 6, Benjamini Hochberg’s test, q < 0.25). Compared to day 0, *Flavonifractor plautii ERR2221305, Helicobacter pylori_BU GCF_000345165*, and *Massilibacterium senegalense GCF_001375675* were increased after 2 or 4 days of GP-SPI intake with *Massilibacterium senegalense GCF_001375675* remaining elevated at day 10 (Fig. 5B; Supplementary Table 6). 10 days of GP-SPI intake increased abundance of *Bilophila wadsworthia 111.35* (Fig. 5B; Supplementary Table 6). After 10 days of GP-SPI intake, *Eubacterium_E hallii ERR1022398* and *Eubacterium_E hallii 14.29* abundances were significantly reduced (Fig. 5B; Supplementary Table 6); however, most genomes were only transiently reduced at 2 or 4 days of GP-SPI intake i.e., *Clostridium_Q symbiosum ERR1022380, Sutterella wadsworthensis_B, Megasphaera sp900066485 ERR1022370*, *Cutibacterium acnes ERR2221340, Helicobacter pylori_BU GCF_000345225, Helicobacter pylori_BU GCF_000345065, Helicobacter pylori_BU GCF_000345605, Helicobacter pylori_BU GCF_000345685, Streptomyces albus GCF_000411495, Micrococcus luteus ERR2221282, Ralstonia pickettii GCF_000227255, Blautia_A obeum ERR1022339*, and *Blautia_A wexlerae ERR2221098* (Fig. 5B; Supplementary Table 6), suggesting most microbes are resilient to the effects of PAC compounds.

After GP-SPI supplementation, the mean abundances of bacterial genomes present in males and females were mostly similar (Supplementary Table 7) with some sex specific differences. *Ralstonia pickettii GCF_000227255* was changed in both males and females, while *Helicobacter pylori_BU GCF_000345265* was changed only in males, and five genomes (i.e., *Cutibacterium acnes ERR2221340, Eubacterium_E hallii 14.29, Helicobacter pylori_BU GCF_000345685, Micrococcus luteus ERR2221282, and Streptomyces albus GCF_000411495*) were changed only in females (Supplementary Table 8, Benjamini Hochberg’s test, q < 0.25)

### GP-SPI supplementation increased relative abundance of one strain of *Akkermansia muciniphila*

Total bacteria detected by qPCR was significantly reduced after 4 days of GP-SPI but recovered by day 10 (Fig. 5C), which was consistent with shotgun metagenome sequencing data showing GP-SPI intake resulted in a temporary suppression of bacterial taxa (Fig. 5A) that may free additional substrate or allow a larger ecological niche within the gut for other microbes to proliferate. qPCR quantification of *A. muciniphila* relative to total bacteria showed a significant increase at day 4 but not at day 10 (Fig. 5C). Since our prior studies showed that GP-SPI suppressed other taxa while increasing relative abundance of *A. muciniphila* within the murine microbiome^5,12^, we further examined *A. muciniphila* genomes. Eight *A. muciniphila* HQMAGs were detected in the metagenomic dataset (Table 3). After 10 days of GP-SPI supplementation no individual *A. muciniphila* strain was increased using the Benjamini Hochberg post hoc test (q > 0.25); however, a secondary analysis using Nemenyi post hoc test suggested an increase in *A. muciniphila 208.2* (*p* < 0.05, Table 3) although only in a subset of subjects (Fig. 5D). Interestingly, nineteen subjects had no detectable levels of *A. muciniphila 208.2* at day 0, but six of these subjects had a detectable increase to this strain after 10 days of GP-SPI supplementation. All but one of the eight subjects with detectable levels of *A. muciniphila 208.2* at day 0 experienced an increase in abundance of *A. muciniphila 208.2* by day 10. These data again demonstrate divergent responses to a common treatment due to the heterogeneity of humans and their microbiomes.

**Table 3:** Fecal abundance of *Akkermansia muciniphila* genomes

	Mean abundance ± SD (%)					
Strain	Day -5	Day 0	Day 2	Day 4	Day 10	*p*-value
9.28	0.0412 ± 0.207^a^	0.0376 ± 0.192^a^	0.0228 ± 0.113^a^	0.0151 ± 0.067^a^	0.0382 ± 0.183^a^	0.57
29.4	0.0098 ± 0.031^a^	0.0178 ± 0.087^a^	0.0214 ± 0.106^a^	0.0163 ± 0.079^a^	0.0158 ± 0.068^a^	0.71
B 49.30	0.0925 ± 0.479^a^	0.0048 ± 0.024^a^	0.0284 ± 0.139^a^	0.0451 ± 0.233^a^	0.0248 ± 0.127^a^	0.90
B 58.31	0.0619 ± 0.309^a^	0.0365 ± 0.184^a^	0.0227 ± 0.113^a^	0.0370 ± 0.188^a^	0.2136 ± 1.08^a^	0.24
62.7	0.0048 ± 0.015^a^	0.0232 ± 0.115^a^	0.0097 ± 0.046^a^	0.0064 ± 0.026^a^	0.4084 ± 2.11^a^	0.06
B 70.12	0.05 ± 0.241^a^	0.0125 ± 0.052^a^	0.0649 ± 0.234^a^	0.0307 ± 0.128^a^	0.0053 ± 0.012^a^	0.97
B 100.14	0.0024 ± 0.011^a^	0.006 ± 0.03^a^	0.0193 ± 0.098^a^	0.1897 ± 0.982^a^	0.0253 ± 0.128^a^	0.03
208.20	0.0085 ± 0.032^ab^	0.0014 ± 0.006^a^	0.0009 ± 0.002^ab^	0.0084 ± 0.039^ab^	0.0103 ± 0.046^b^	0.006

Data are mean ± standard deviation (SD). Friedman’s test was performed to calculate differences across the repeated measures (*p*-value), followed by the Nemenyi post hoc test for pairwise comparisons.

Different subscript letters (a, b) denote statistically significant differences in mean strain abundance between days (*p* < 0.05).

### Guild-based analyses

Bacteria in the gut ecosystem are not independent but rather co-exist as functional groups called guilds, which interact to affect host health^51^. To identify potential guilds, we explored the co-abundance relationships among 1517 prevalent and dominant genomes that were shared in at least 20% of the samples and accounted for ~98.6% of the total abundance. The 1517 genomes were grouped into 172 distinct guilds (Supplementary Table 9, guild member list). As observed with genome-level beta diversity, guild-level beta diversity analysis also showed that longitudinally collected fecal samples from the same participant clustered together with significant inter-individual variation (Supplementary Fig. 5F). Despite apparent clustering of microbial communities in male and female subjects at the different time points (Supplementary Fig. 5G), GP-SPI intake did not alter guilds in a sex-specific manner (Supplementary Table 10).

aPCoA plots and subject-stratified PERMANOVA testing on Bray-Curtis dissimilarity for all participants showed significant separation of guild-level microbial composition after 5 days of SPI supplementation (day -5 vs. day 0) and after 2 days of GP-SPI supplementation (day 0 vs. day 2, Fig. 5E, F; Supplementary Table 5). Changes to guild structure was largely driven by male subjects, as males, but not females, showed significant changes in guild composition (day 0 vs. day 2 and day 4, Fig. 5F). Bray-Curtis analyses by guild-level showed that bacterial communities reverted to day 0 profiles after 10 days of GP-SPI supplementation, though changes were sustained at the genome-level (Supplementary Table 5), suggesting bacterial guilds adapted to GP-SPI intake despite sustained changes to some low abundance taxa. Compared to day 0, Jaccard dissimilarity at the genome-level showed separation after 2 and 4 days of GP-SPI intake, but this difference was not sustained at day 10 (Supplementary Table 5). Jaccard dissimilarity at the guild-level showed no differences between study days (Supplementary Table 5), indicating that presence/absence changes were not prevalent among microbial guilds. Therefore, GP-SPI appeared to alter abundances of existing genomes or guilds more than the membership of the gut microbiota.

Five guilds were differentially affected by SPI and/or GP-SPI supplementation (Fig. 5G, Supplementary Table 9). GP-SPI supplementation increased abundance of guilds 87 and 142 on day 2, but significance was lost at later time points (Fig. 5G). Guilds 87 contained *Adlercreutzia equolifaciens 205.21, Anaerococcus sp001182725 GCF_001182725, Anaerovoracaceae ERR2221283, Butyricimonas synergistica GCF_900091675, Lawsonibacter sp000177015 GCF_000177015, MS4 sp000752215 GCF_000753235* and guild 142 contained *Acutalibacteraceae 78.25, Agathobaculum butyriciproducens 77.5, Agathobaculum butyriciproducens 78.2, Alistipes finegoldii 211.1, Bilophila wadsworthia 111.35, Clostridium_M asparagiforme GCF_000158075, Collinsella 76.20, Eggerthellaceae 111.7, Eubacterium_I ramulus ERR1022349, Eubacterium_I ramulus GCF_000469345, Gordonibacter pamelaeae ERR2221387, Holdemania sp900120005 101.6*, and *Odoribacter splanchnicus 78.11*. Of the genomes in increased guilds, only *Bilophila wadsworthia 111.35*, a bile-resistant sulfidogenic bacteria^52,53^, was significantly modulated over the time period (q < 0.25), with a significant reduction after the SPI intake phase, and increases and decreases over the GP-SPI phase, but ultimately reverting to an insignificant increase in abundance by day 10 (Fig. 5B).

Guild 139 was increased by SPI and remained higher throughout GP-SPI supplementation (Fig. 5G), and contained *Agathobaculum sp900291975 ERR1022441, Citrobacter freundii ERR2221352, Clostridium_M bolteae 74.6, Clostridium_M citroniae GCF_001078435, Clostridium_M ERR171262, Clostridium_Q symbiosum ERR171274, Dorea sp000509125 ERR1203939, Faecalicatena contorta_B GCF_001244405, Faecalicatena glycyrrhizinilyticum 74.2, Hungatella ERR171272, Hungatella ERR2230151, Lachnospiraceae 73.5, Lachnospiraceae 75.8, Raoultibacter massiliensis 75.2, and Roseburia inulinivorans 75.7*. Of the genomes within guild 139, only *Clostridium_M bolteae 74.6* was significantly increased (Fig. 5B), though this increase is attributable to SPI and not GP intake.

GP-SPI supplementation reduced guilds 53 and 166 on days 2 and 4, but in both cases guild levels recovered by day 10 (Fig. 5G). Guild 53 included *Clostridium_M clostridioforme ERR2221183, Clostridium_M clostridioforme ERR2221212, Clostridium_M clostridioforme ERR2230050, Clostridium_Q symbiosum ERR1022380, Cutibacterium acnes ERR2221340, Escherichia coli GCF_000164495, Escherichia coli GCF_000164615, Escherichia coli_D GCF_000164315, Escherichia flexneri GCF_000164335, Escherichia flexneri GCF_000164555, Flavonifractor plautii ERR2221213, Flavonifractor plautii GCF_000239295, Megasphaera sp900066485 ERR1022370, Micrococcus luteus ERR2221282, Streptomyces albus GCF_000411495*, and *Sutterella wadsworthensis_B ERR1203961*. Several genomes in guild 53 were significantly reduced by GP-SPI, including *Clostridium_Q symbiosum ERR1022380, Micrococcus luteus ERR2221282, Streptomyces albus GCF_000411495*, and *Sutterella wadsworthensis_B ERR1203961 (*Fig. 5B*)*. Guild 166 contained a cluster of nine *Streptococcus* spp. i.e., *ERR2221353, ERR2221360, gordonii GCF_000161955, infantarius GCF_000154985, parasanguinis ERR2221190, parasanguinis_B ERR2221175, salivarius ERR2221359, sp000411475 GCF_000411475*, and *timonensis GCF_900095845*.

### GP-SPI-supplementation did not significantly modify the abundance of functional genes within the gut microbiome for metabolism of polyphenols, bile acids, or short chain fatty acids

Functional annotation of the fecal microbial genomes was conducted to identify enzymes involved in polyphenol, bile acid, and short chain fatty acid (SCFA) metabolism to determine if abundance of metabolites could be affected via altered abundance of metabolizing bacteria (Supplementary Table 11). Supplementation with SPI (day -5 to day 0) resulted in increased gene abundance of BA metabolizing genes (*baiA, baiCD, baiF, baiE, baiH*) that was sustained after 2 days of GP-SPI supplementation; however, after 4 and 10 days of GP-SPI intake, gene counts reverted to pre-supplementation levels (Supplementary Table 11). The *baiG* gene, encoding a bile acid transporter allowing bacterial uptake of CA, CDCA, and DCA^54^, was significantly decreased after 2 and 10 days of GP-SPI intake; gene abundances for *7α/β-hsdh, bsh*, and *bail* were unaltered by supplementation (Supplementary Table 11). The terminal enzyme for butyrate production (*buk*) was increased after 10 days of GP-SPI supplementation, but other SCFA synthesis genes were unchanged (*but, 4-hbt, atoA, atoD, ftfL*) or only transiently changed (*pct, scpC*; Supplementary Table 11). Levels of polyphenol metabolism genes were not significantly changed (Supplementary Table 11). These functional gene data are consistent with microbial abundance not driving the changes in BA levels in serum or polyphenol metabolite levels in serum or urine samples.

### GP-SPI induced transient changes to gut bacterial proteins

Metaproteomic analysis on fecal bacteria from day –5, 0, 2, 4, and 10 was performed to gain insight into how SPI and GP-SPI supplementation affected bacterial function. Out of 6513 identified proteins 6161 were microbial, of which 4153 aligned with the predicted proteins from metagenomics with 90% identity.

**Fig. 6:**
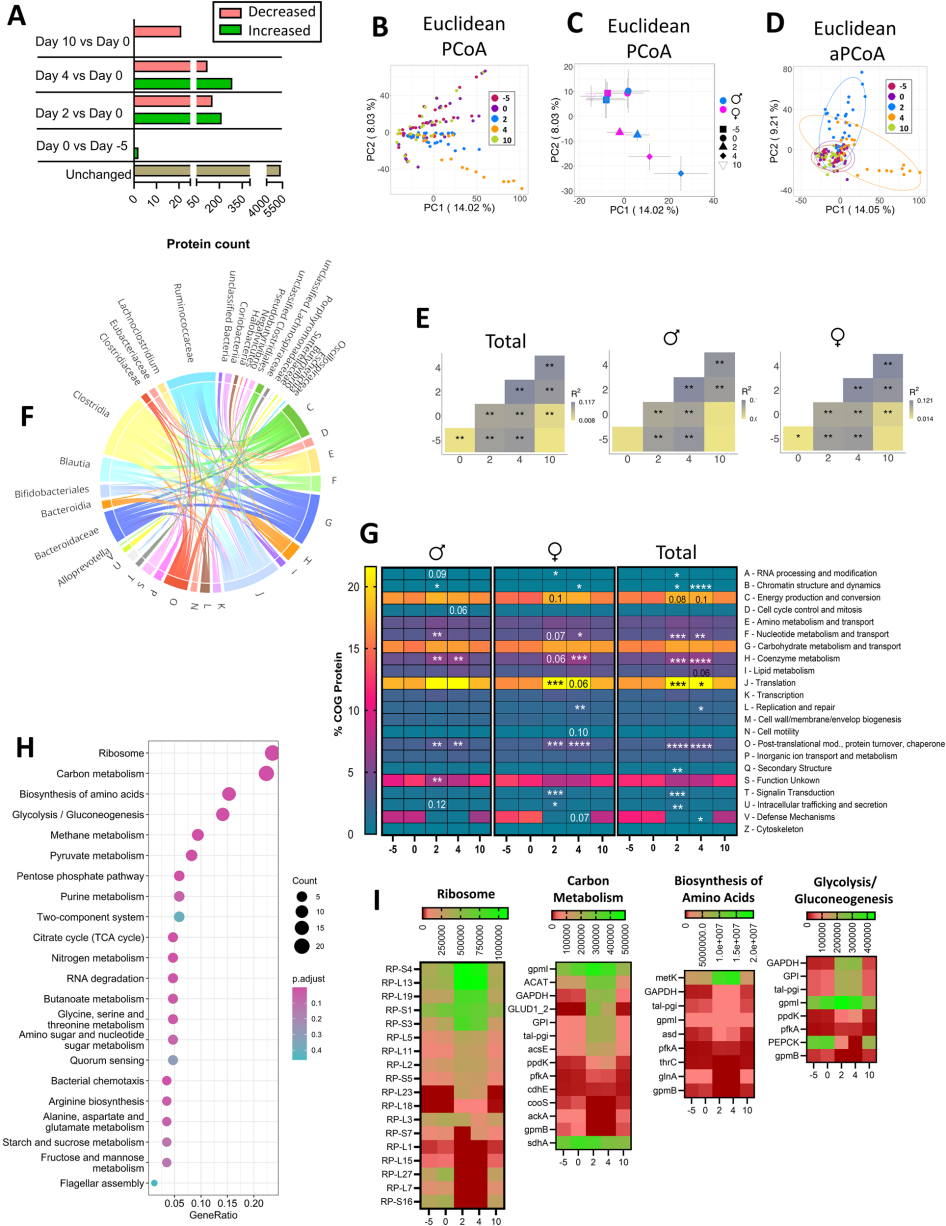
GP-SPI induced transient changes to gut bacterial proteins. **A** Number of up- or down-regulated fecal microbial proteins after 5 days of SPI-supplementation (Day 0 vs Day –5), or after GP-SPI-supplementation (Day 0 vs. Day 2, 4, or 10), based on a threshold of q ≤ 0.25. **B** PCoA plot of microbial proteins based on Euclidean distance, where each dot represents a subject. **C** PCoA plot of microbial proteins based on Euclidean distance, presented as mean ± SEM of male and female groups at each timepoint. **D** Adjusted PCoA (aPCoA) plot based on Euclidean distance, where each dot represents a subject. **E** Subject stratified PERMANOVA test results based on Euclidean distance of total participant pool, males, and females. **F** Chord diagram displaying bacterial source of the COG proteins changed by GP-SPI on day 2, 4, or 10 vs. day 0, threshold of q ≤ 0.25. **G** Heatmap showing distribution of COG (A-V) categories (%) over days –5, 0, 2, 4, and 10 of the study. Difference was determined by Freidman test followed by Dunn’s posthoc test comparing each timepoint to day 0. *p < 0.05; **p < 0.001; ***p < 0.0001, ****p < 0.000001. **H** KEGG Enrichment bubble plots showing pathways (with 2 or more proteins) affected over 2 or more timepoints during the GP-SPI supplementation (i.e., Day 0 vs. Day 2, 4 or 10). The vertical axis represents the enriched KEGG classification (fold >1.5, *p* < 0.05). The horizontal axis is the rich factor, representing the ratio of the number of differentially expressed proteins to those in the entire KEGG pathway. The size of the circular area represents the number of differentially expressed proteins, and the circular color represents the enrichment adjusted *p* value (p.adjust) of the differentially expressed proteins under the KEGG classification. **I** Heatmaps of the top 4 enriched canonical pathways. Gradient towards green are up-regulated proteins, and towards red are down-regulated proteins.

A total of 215 and 285 microbial proteins were upregulated while 155 and 122 were downregulated after 2 and 4 days of GP-SPI intake, respectively; after 10 days of GP-SPI supplementation, 21 microbial proteins were decreased, and none were increased (Fig. 6A**;** See Supplementary Table 12 for a list of identified microbial proteins). Unadjusted PCoA based on Euclidean distances was used to visualize microbial protein profiles of each participant on days –5, 0, 2, 4, and 10 (Fig. 6B). While SPI alone did not alter bacterial proteins (day –5 vs. day 0), significant changes were transiently detected after 2 and 4 days of GP-SPI intake, but by day 10 of GP-SPI supplementation the bacterial protein profiles returned to day –5 and day 0 profiles (Fig. 6B, Supplementary Table 13). Unadjusted PCoA of subjects grouped by sex showed that microbial protein profiles were similar between males and females for each study day (Fig. 6C). Similarly, after inter-subject variation adjustment, aPCoA plots showed transient alteration in microbial proteins after 2 or 4 days of GP-SPI intake (Fig. 6D). Subject stratified PERMANOVA results showed significant difference between microbial protein profiles of all participants at all timepoints, except between day –5 and day 10 (Fig. 6E, Supplementary Table 13). A difference between subject stratified microbial protein profile at day 0 vs. day 10 was observed in females but not males (Fig. 6E). The abundances of many individual microbial proteins were changed over the intervention period (Supplementary Table 14), but there were no significant differences between protein abundances in males and females (Supplementary Table 14).

Changes to microbial proteins were further investigated by clustering the identified proteins into functional groups. Among the detected proteins, 6106 were assigned to 22 clusters of orthologous group (COG) categories, of which 6029 had bacterial taxonomic assignment according to MGnify (https://www.ebi.ac.uk/metagenomics). Most proteins altered by GP-SPI intake originated from members of the Firmicutes phylum (i.e., families Clostridiaceae, Eubacteriaceae, Ruminococcaceae, Lachnospiraceae, and Oscillospiraceae, classes Negativicutes, Clostridia, and genera *Blautia*, *Butyrivibrio*, and *Lachnoclostridium;* Fig. 6F). COG categories were significantly changed after 2 and/or 4 days of GP-SPI supplementation, but significance was lost by day 10 in males and females (Fig. 6G) suggesting bacterial resilience. Proteins that were up-regulated by GP-SPI intake were in categories with functions relating to RNA processing and modification, chromatin structure and dynamics, energy production and conversion, nucleotide metabolism and transport, coenzyme metabolism, replication and repair, translation, post-translational modifications/protein turnover, secondary structure, signaling, signaling transduction, and intracellular transport (Fig. 6G). Notably, only defense mechanisms (COG category V) were down-regulated by GP-SPI at day 4 (Fig. 6G), which may provide mechanistic insight to the antimicrobial effects of GPs. However, after removing subjects with outlier values at one or more timepoints, the % reduction in COG category V was not as distinct in total subjects (Freidman’s test followed by Dunn’s post hoc test, day 0 vs. day 4, *p* = 0.18).

We next investigated KEGG orthology (KO) to gain insight into the effect of SPI or GP-SPI supplementation on more specific bacterial activities. Of the detected microbial proteins, 4794 were assigned KO IDs. Several proteins with the same KO assignment increased and decreased, suggesting the same proteins expressed by different bacteria responded to GP-SPI supplementation differently (Supplementary Table 12), possibly due to different post-translational modifications or other regulatory mechanisms in different strains/species.

We screened KOs affected by SPI or GP-SPI, as outlined in Supplementary Fig 8 for analyses presented in Fig. 6H, I. SPI supplementation increased only 2 proteins, lactose transport (lacE) and acetyl-coA c-acetyltransferase (ACAT), which mapped to 18 canonical pathways using KEGG Mapper. GP-SPI altered expression of 160 proteins with available KOs, which were mapped to 107 canonical pathways (see Supplementary Table 15 for all mapped KOs). KEGG enrichment analyses detected the pathways most affected by GP-SPI supplementation, and the top 4 pathways were related to the ribosome, carbon metabolism, biosynthesis of amino acids, and glycolysis/gluconeogenesis (Fig. 6H). Different ribosomal EF-Tu subunits, which facilitate protein translation and aid in bacterial binding to host cells^55^, were increased or decreased on days 2 and 4, but all were down-regulated after 10 days of GP-SPI supplementation (Fig. 6H). After 2 and 4 days of GP-SPI supplementation, expression of enzymes involved in initial steps of glycolysis and the reductive pentose phosphate cycle were increased, but then decreased at day 10 (Supplementary Table 16). GP-SPI altered production of bacterial ATP-binding cassette (ABC) transporters for peptides and saccharides (Supplementary Table 16). Reductions to ABC transporter, NisF, over day 2 and 4 (Supplementary Table 16), may also serve to protect select bacteria from increased levels of bacteriocin or nisin^56^. While the category of defense proteins was reduced by GP-SPI (Fig. 6G), there was a transiently increased expression of bacterial chemotaxis and quorum sensing proteins (Supplementary Table 16) that have roles in bacterial defense. Selective upregulation of defense proteins may be a mechanism by which certain bacteria prevailed while others were downregulated. GP-SPI intake altered proteins of the two-component regulatory system, specifically by increasing FliC, a protein important for flagellar assembly and bacterial growth^57^, and reducing heat shock protein 90 (Hsp90), a protein needed for cell division via interactions with a tubulin homolog^58^ (Supplementary Table 16). Changes to proteins within the two-component system suggest GP-SPI altered bacterial responses to changed environmental conditions and regulation of proliferation. Although most changes to microbial protein levels reverted to pre-GP-SPI supplementation levels by day 10, a few changes were sustained for 2 consecutive timepoints including day 10 in some, but not all bacteria, i.e., reductions to succinate dehydrogenase (sdhA; K00239), biopolymer transport protein (exbB; K03561), and hydroxylamine reductase (hcp; K05601), pyruvate-ferredoxin/flavodoxin oxidoreductase (por; K03737) and raffinose/stachyose/melibiose transport system substrate-binding protein (msmE; K10117; Supplementary Table 16). The physiological consequences of the few sustained protein changes resulting from GP-SPI remain to be investigated, but we predict that GP-SPI effects on expression of bacterial protein may shift bacterial functions that favor survival of select taxa.

### GP-SPI induced transient changes to host proteins

Although the protocol was optimized for extraction of fecal bacterial proteins, 352 human proteins contained in the fecal samples were also obtained. The PCoA plot showed that, as with bacterial proteins, host proteins showed great inter-subject variability and proteins clustered together based on supplementation day rather than sex (Supplementary Fig. 6A, B). Comparison of individual proteins between males and females also did not differ by a threshold of q ≤ 0.25 (Supplementary Table 17). After correcting for inter-subject variables, the aPCoA showed that compared to day –5 or day 0, host proteins were significantly changed on days 2 and 4, but these changes reversed on day 10 (Supplementary Fig. 6C). Based on subject stratified PERMANOVA testing, host protein profiles were different at all measured timepoints for the total group of participants and when separated according to sex (Supplementary Fig. 6D). SPI supplementation increased 2 host proteins, human intelectin-1 (ITLN1) and immunoglobulin kappa variable 3-20 (KV320), while GP-SPI supplementation differentially altered 12 host proteins at more than 2 consecutive timepoints when compared to day 0 i.e., chymotrypsinogen B1 (CTRB1), leucine rich repeat and sterile alpha motif containing 1 (LRSAM1), lactate dehydrogenase A (LDHA), DOP1 leucine zipper like protein A (DOPEY1), ubiquitin (UBB), cluster of differentiation 109 (CD109), immunoglobulin Kappa Variable 2D-24 (KVD24), tropomyosin 2 (TPM2), maestro heat-like repeat-containing protein family member 7 (MROH7), ATP synthase subunit alpha (ATP5A1), fructose-bisphosphate aldolase A (ALDOA), and proteoglycan (PRG2) (Nemenyi’s posthoc test, q < 0.25; Supplementary Table 18).

STRING 11.5 was able to assign 11 out of 12 host proteins affected by GP-SPI, with 3 edges, a node degree of 0.545 and average clustering coefficient of 0.273 and protein-protein interaction (PPI) enrichment *p*-value of 0.133, which suggests that the changed proteins are not highly connected. Changes in the detected human proteins included those with known roles in digestion (CTRB1), metabolism (LDHA, ALDOA, ATP5A1) and immunity (LRSAM1, CD109 PRG2), and other miscellaneous functions like protein shuttling and muscle contractions. Of these, TPM2, CD109, LDHA, ATP5A1, ALDOA, and UBB are known to be expressed in the intestine. ATP5A1, LRSAM1, LDHA, UBB, CD109, TPM2, and ALDOA are related to extracellular exosomes based on identification in the online ExoCarta database^59^. Based on functional enrichments detected by STRING, GP-SPI supplementation upregulated expression of host proteins (ALDOA, and LDHA) that are commonly co-expressed and are involved in glycolysis (Supplementary Fig. 6E). These findings provide insight into potential effects of GP-SPI on host functions in the gut and warrant further investigation.

### Correlational analysis

**Fig. 7:**
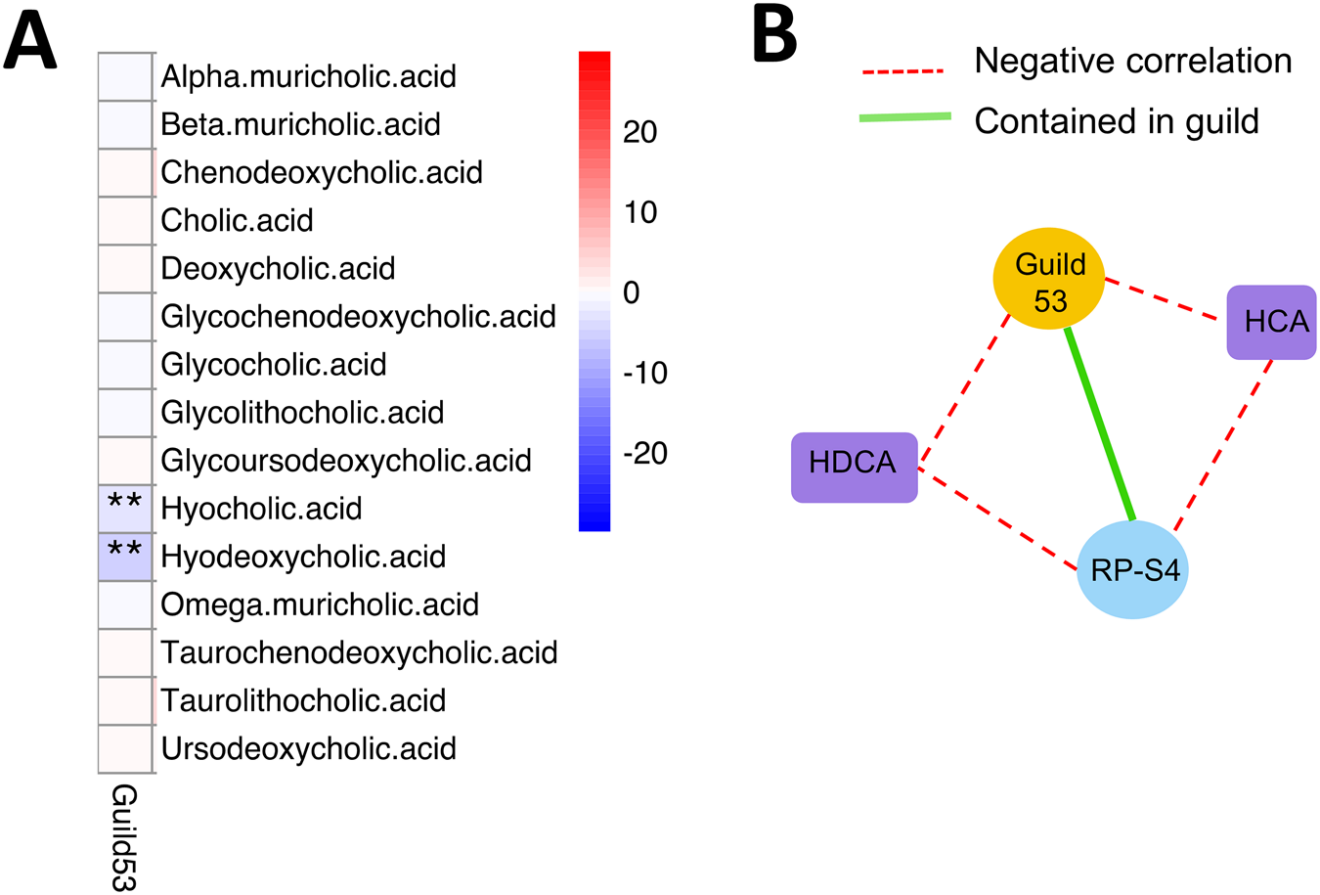
Correlational analyses. Correlations found between (**A**) serum BA and bacterial guilds, and between (**B**) serum BA, guilds, and bacterial protein, small subunit ribosomal protein S4 (RP-S4; K02986), as determined by Maaslin2. **p < 0.005.

Correlational analysis was performed to integrate the metabolomics, proteomics, and metagenomics datasets obtained before and after GP-SPI supplementation to better understand which fecal bacterial guilds were responsible for producing measured metabolites. Significant correlations between changed bacterial guilds, bacterial proteins, and polyphenol-derived metabolites were not detected. Reduced abundance of guild 53 correlated with increased concentrations of HCA and HDCA in serum (Fig. 7A), raising the possibility that these bacteria metabolize these BAs to some other form. Guild 53 contained small subunit ribosomal protein S4 (RP-S4), which was negatively correlated with both serum HCA and HDCA levels, suggesting a potential connection between these BAs and this bacterial protein within guild 53 (Fig. 7B).

## Discussion

The biological signature of GP-SPI supplementation involves targeted changes to bacterial guilds, proteins, and metabolites. Food intake data indicated high protocol compliance, specifically the restriction of PAC-rich foods during the study period so any effects of PACs provided by the GP-SPI intervention could be isolated. While abstaining from PAC-rich foods, subjects consumed 40 g/day of GP-SPI delivering 1.6 g of PAC per day, which is a challenging dose to obtain from diet alone as it is 3.5 times higher than the estimated daily intake for a Mediterranean diet^33^. Based on CMP data and participant self-reporting, this higher than usual PAC dose over the short 10-day time frame did not result in adverse effects. Clinical studies reported that subjects supplemented with 100 mg–2000 mg/day of grape seed extract (in addition to polyphenols consumed in foods) for 2 – 16 weeks had beneficial effects on blood pressure^60^, suggesting a wide window of therapeutic benefit and safety for PAC compounds. Here we aimed to replace dietary PACs with 1.6 g of PACs provided by GP-SPI and found that although changes to the gut microbiome, BA, and PAC-metabolite profiles occurred rapidly after 2 or 4 days of GP-SPI intake, only metabolite changes were sustained after 10 days as most microbiome and metaproteome changes reverted to baseline profiles by day 10.

HCA levels are inversely related to fasting and postprandial glucose levels^47,48^. Two prospective cohort studies found that HCA levels were lower in patients with obesity, pre-diabetes, and diabetes, and that increases to HCA levels after gastric bypass surgery were correlated with remission of diabetes^48^. In both healthy mice and diabetic mouse models, oral administration of HCA improved oral glucose tolerance and increased active GLP-1 in circulation^47^. In enteroendocrine L cells, HCA was found to activate TGR5, resulting in GLP-1 secretion while also inhibiting FXR, leading to increased transcription of the GLP-1 precursor, proglucagon^47^. Our observed increase in serum HCA and the trending increase in HCA species (Fig. 4C) was consistent with the lower fasting blood glucose measured in participants after GP-SPI supplementation (Table 2). Serum HCA species made up 15% of the total BA pool in our targeted LC-MS quantification, a higher proportion than previous reports (i.e., 2–3%), which may be due to differences in LC-MS instrumentation or other parameters of analysis; our serum BA recovery was 87–91%. The BA changes induced by GP-SPI in human participants were not the same as those observed in prior murine experiments^12,61^, and may be due to species differences.

Previous murine studies have suggested that the BA profile induced by GP-supplementation could improve glucose metabolism and reduce ceramide synthesis by reducing FXR activation in the intestine^12^. GP-supplementation was also associated with reduced mRNA markers of FXR activation in hepatic tissue of *db/db* mice, along with increased hepatic markers for BA synthesis and altered BA transport^61^, which may be attributable to bioavailable PAC metabolites. Reductions to levels of FXR agonist CDCA in serum from GP-SPI may also have metabolic implications as this BA was shown to be higher in serum of diabetics, and has been positively correlated with BMI, glycated hemoglobin, triglycerides, and LDL cholesterol^62^. UDCA, a BA increased in circulation from GP-SPI supplementation, was associated with improved liver function in patients with obesity and liver dysfunction^63^, and is approved by the US Food and Drug Administration for treatment of primary biliary cholangitis. Several studies have reported that polyphenols promote metabolic benefits in association with altered BA levels and/or signaling in the intestine and liver^21^, therefore it is likely that GP-supplementation influences BA signaling in these tissues.

Concentrations of microbial polyphenol metabolites DHPV, 3-PPA, pCA, VA, DAT, and 3-HPA increased in fecal content during the GP-SPI-phase, although only DHPV is a direct metabolite of B-type PAC dimers^40^. While these 6 metabolites were increased at some point in fecal samples during GP-SPI supplementation, only DHPV, 3-PPA, *p*CA, and 3-HPA were detected at increased levels in serum or urine, suggesting selective bioavailability of these compounds. Such metabolites may confer bioactivities within the intestine or in circulation. Previous preclinical research has shown potential bioactivities of these PAC-metabolites, such as DHPV-induced improvement to glucose metabolism in muscle cells^64^, pCA-induced inhibition of adipogenesis in fat cells^65^, 3-PPA-induced enhancement of the intestinal epithelial barrier^66^, and 3-HPA-induced reductions to arterial blood pressure^67^. Therefore, diets rich in PAC may confer metabolic health benefits via such microbially derived PAC metabolites. A preclinical study has shown that oral supplementation with DAT protected mice from influenza, and differences in the gut microbiota and production of DAT in human gut microbiota was hypothesized to be a factor in the heterogeneous response to influenza infection^68^. In this study, DAT levels were only increased in the first days of GP supplementation. Furthermore, levels of DAT and other metabolites showed great heterogeneity across subjects. Such results could be due to differences in individual gut microbial communities and regulation of absorption and excretion across participants in this study. Our findings support the need for a personalized approach to investigating dietary interventions that considers the metabolic capabilities of unique gut microbiomes and host genetic polymorphisms, which together may result in differential responses to dietary polyphenols^46,69^.

Based on prior reports, we expected to detect polyphenols in serum, urine, and feces up to 24 h after supplementation^43^. Future studies which measure PAC-metabolite levels over several timepoints and consider the proportion of free and conjugated forms are warranted to confirm bioavailability of compounds due to their rapid rates of excretion^43^. BA profile is also time-dependent, as synthesis is influenced by circadian rhythms^70^. In this study, serum was collected in the mornings in fasted subjects therefore time was not a variable for serum profiles; however, it is important to note that the timing of fecal and urine collection by individual participants was variable throughout the day.

Our shotgun sequencing data were not mirrored by 16S rRNA amplicon sequencing results (data not shown), which emphasizes the importance of strain level analyses compared to lower resolution methods. Bacterial changes in this study were not sustained, therefore it is unclear what the metabolic implications of these changes may be. Nonetheless, there were some noteworthy changes, for example a probable increase in fecal *A. muciniphila 208.2*, which warrants further investigation. Mice supplemented with PAC-rich extracts from grape^5,6,12,71^, cranberry^72^, camu camu^73,74^, or apple^75^ showed increased relative abundance of *A. muciniphila*. In C57BL6/j mice the GP-induced increase in *A. muciniphila* relative abundance was hypothesized to be due to GP-mediated suppression of other gut bacteria^6^. *A. muciniphila* abundance was shown to be lower in subjects with obesity^76^. A clinical study showed that overweight or obese subjects supplemented with heat-killed *A. muciniphila* for 3 months had attenuated symptoms of metabolic disease^29^, confirming pre-clinical work showing *A. muciniphila* cell surface protein Amuc_1100 contributed to metabolic benefits^28^. Importantly, *A. muciniphila* secretes an 84 kDa protein P9, which binds intercellular adhesion molecule 2 (ICAM-2) to promote secretion of the incretin glucagon-like peptide-1 (GLP-1)^77^. Therefore, dietary interventions that increase or maintain *A. muciniphila* within the gut microbial community may promote glucose homeostasis. Two other clinical studies reported increased *A. muciniphila* after subjects were supplemented with grape powder (161 mg total polyphenols) for 4 weeks^35^ or sweetened dried cranberries (93.3 mg PAC) for 2 weeks^34^. It is not clear whether *A. muciniphila* is integral to polyphenol-induced benefits, as displayed by a rodent study showing that camu camu (62.5 mg/kg) induced a bloom of *A. muciniphila* and decreased non-HDL cholesterol and free fatty acids, while a higher dose (200 mg/kg) did not induce such a bloom, but still resulted in several metabolic improvements, i.e., reduced weight and fat mass gain and reduced hepatic steatosis^74^.

Reduction of *H. pylori*, a bacterium associated with stomach-ulcers and obesity^78^, has been recapitulated in several polyphenol-intervention clinical studies based on a recent review^79^. DCA was shown to be antibacterial to *H. pylori*^80^, therefore GP-induced increase to fecal DCA may have contributed to its transiently reduced abundance. Reductions to several *H. pylori* strains after GP-SPI supplementation could be due to reduced cellular adhesion, as reported in a previous intervention using apple peel polyphenols^81^.

We hypothesize that the gut microbiome adapted to the antibacterial effects of GPs and metabolites. Results to metaproteomic analysis in this study revealed that expression of several bacterial proteins associated with proliferative success were temporarily altered by GP-SPI supplementation, including bacterial proteins involved in two-component system, in bacterial chemotaxis, stress and protein misfolding, monobactam biosynthesis, and ribosomal components. GP-SPI-induced changes to proteins involved in metabolism, synthesis and transport of carbohydrates and amino acids likely also affected bacterial growth and replication. Metaproteomic analysis did not show changes to the abundance of bacterial proteins involved in polyphenol or BA metabolism, and thus we hypothesize that changes to concentrations of these metabolites was due to changes in available substrate rather than altered expression of bacterial proteins. Further research is warranted to understand how bacterial protein activity is affected, in addition to protein concentrations.

GP-SPI-induced changes to the abundance of individual guilds and genomes appeared to be independent of sex; however, sex-specific responses were detected in changes to BA and PAC metabolite profiles. There are known sexual dimorphisms between absorption of polyphenols^82^, and their regulation of gut microbiota^83^. Thus, clinical studies with power to differentiate sex-specific responses are needed. Additionally, considering food intake as a variable is important because of the effects of dietary patterns on gut microbiota^84^, and synergistic effects of fiber on polyphenol absorption^85^ and health outcomes^86^. Although differences between sexes and dietary patterns were considered in our analyses, the limitations of the present study were: (1) inclusion of only healthy subjects, (2) relatively small sample size, and (3) using a longitudinal design. Nevertheless, to our knowledge, this is the first clinical study to suggest that dietary intake of GPs (of which ~78% was B-type PACs), can promote HCA, a glucoregulatory BA that is protective against metabolic disorders^47,48^. Although significant changes to metabolic blood values were detected, all values remained within the normal range in healthy participants. Research on individuals with impaired glucose metabolism is needed to better understand the clinical significance of these changes. It remains to be determined whether GPs can increase circulating HCA species to improve glucoregulation in human subjects with impaired fasting glucose. Human fecal transplant studies to gnotobiotic mice may provide insight to the role of a GP-modulated gut microbiota to changes in metabolite profile. While the present longitudinal design was useful for comparing changes of each participant to the pre-intervention state, a randomized cross-over study design in a larger group of subjects is needed to confirm whether GPs can promote beneficial changes in HCA and blood glucose via suppression of bacterial guild 53.

## Methods

### GP-SPI production and biochemical characterization

Using previously described methods adapted to pilot scale equipment in the Rutgers Food Science pilot plant facility, GPs were extracted from frozen Concord grape pomace (Welch Foods Inc, Concord, MA, USA) and complexed to SPI (PRO-FAM^®^ 955, ADM, Decatur, IL) to produce GP-SPI complex^5,6,25^. Ultra performance liquid chromatography (UPLC) coupled to high resolution mass spectrometry (MS) was used to characterize major polyphenol species in the GP extract. Supplementary Fig. 7 shows retention times and spectrometric characteristics of major GPs in the extract, which included catechin/epicatechin monomers, B-type PAC dimers, trimers, dimer gallates, trimer gallates, tetramers, and pentamers, as well as stilbenes and oligostilbenes. The Folin-Ciocalteau assay using gallic acid as standard^87^ was used to quantify the total polyphenols in the GP extract, and using the 4-dimethylaminocinnamaldehyde (DMAC) assay with PAC B2 (Cayman Chemicals, catalog # 19865) as standard^25,88^ it was determined that 78% of the total GPs were PAC compounds. A calculated amount of SPI was added to the GP extract followed by rotary evaporation (Rotavapor R-187, Büchi) and convection oven drying at 40 °C (Power O Matic-60, Blue M Electric Company, Blue Island, IL, USA) for an average of 20 h to produce a GP-SPI powder containing 5% total GPs and < 15% moisture. A sample of GP-SPI powder underwent the same battery of tests routinely performed for Rutgers University dining halls and was found to be microbiologically safe (Supplementary Table 19). The total calories, carbohydrate, fat, protein, and fiber content of SPI and GP-SPI powders were determined by Medallion Labs (Supplementary Table 20). 20 g doses of GP-SPI or SPI were packaged into individual 1-ounce zip pouches (Pack Plus Converting Co., Chino, CA) that were heat sealed and stored in a 4 °C refrigerator until use. Each 20 g dose of GP-SPI contained 1 g of GPs, of which 0.8 g (80%) were PACs.

### Inclusion/exclusion criteria for enrollment

Participants were recruited via flyers and emails circulated on Rutgers University campuses in New Brunswick, New Jersey from June - December 2019. Inclusion criteria were: (1) healthy as assessed based on a medical evaluation including a fasting comprehensive metabolic panel (CMP) test with values in normal range, medical history, and not taking medications; (2) adults between 18 and 35 years; (3) body mass index (BMI) of 18.5–29.9 kg/m^2^; (4) have at least one bowel movement per day; and (5) capable of giving written informed consent, which includes compliance with the requirements and restrictions listed in the consent form. Exclusion criteria were: (1) history/current cancer, rheumatoid arthritis, immunologic, renal, hepatic, endocrine, neurologic or heart disease, hypertension, diabetes, gastrointestinal dysfunction, or CMP test results showing values outside of normal range; (2) cannot provide written informed consent; (3) exposure to any experimental agent or procedure within 30 days of study; (4) pregnancy or breast-feeding; (5) taking dietary supplements; (6) current smoker or have smoked within previous 6 months; (7) taking medications regularly (prescription, over the counter, supplements etc.); (8) treated with antibiotics during the past 6 months; or (9) have an allergy to soy or grapes. After explanation of the study and signing of the consent form, a fasting blood draw was scheduled with the study nurse for CMP test, then CMP results were reviewed by the study physician, and participants were allowed to continue with the study intervention only if their CMP results were within normal ranges. Participants were withdrawn after enrollment if they became ill, began any medications, could not provide complete sample/data sets, or did not comply to the study protocol.

### Study design

Subjects were instructed to first consume 20 g of SPI, two times per day (once before breakfast and once before dinner) for 5 days. The 5-day SPI phase of the study served to acclimate participants to SPI and during this period they washed out of PAC-containing foods. All participants were provided with a list of PAC-free foods they could consume freely, PAC-rich foods to abstain from, and low-PAC foods that they could consume in moderation during the course of the study (Supplementary Table 21). The SPI phase was followed by a 1 day break (day 0) where no supplement was consumed. Participants were instructed to then consume 20 g of GP-SPI, two times per day (once before breakfast and once before dinner) for 10 days, for a daily dose of 2 g total polyphenols of which 1.6 g were PAC (Fig. 2A).

Participants were given a personal-size blender along with suggestions for consuming the SPI or GP-SPI powders (e.g., smoothie recipes). Fecal and urine samples were collected at baseline (Day -5), after the SPI phase (Day 0, before the GP-SPI phase), and after 2, 4, 6, 8, and 10 days of GP-SPI consumption (Fig. 2A) using supplied collection containers. Fasting blood samples were drawn by the study nurse in the morning (8–9 AM) of days 0 and after 10 days of GP-SPI-supplementation (Fig. 2A). Serum was separated by incubating blood for 30-min at room temperature followed by 10 min centrifugation at 12,000 rcf at 4 °C. Participants were provided with paper toilet seat accessories (CAT# OM-AC1, DNA Genotech Inc. ON, Canada) for stool capture, screw-cap tubes with scoops (CAT# 80.623.022, Sarstedt AG & Co, Germany) filled with 5 mL of 95% ethanol for fecal collection. Urine was collected directly into a sterile urinalysis cup (CAT# 90GRN53-1000, ThermoScientific). Participants were provided with opaque carrying cases to hold fecal and urine samples in their refrigerators and were instructed to bring them to the laboratory within 24 h of collection where they were aliquoted into Eppendorf tubes. All samples were stored at –80 °C until analysis.

During the 17-day intervention period, participants took photographs and wrote short descriptions of all the food and drink that they consumed during the study period and sent this information to study coordinators via WhatsApp (Facebook, Inc.). These digital food diaries were used to estimate daily nutrient intake using Food Processor Analysis Software (subscription purchased from ESHA Research, Salem, OR, USA). Participants tracked their stool characteristics using the Bristol stool scale and were asked to record if they were menstruating (chart used provided in Supplementary Table 22).

The primary measure was to evaluate the effect of GP-SPI intake on gut microbiota with specific interest in relative abundance of *Akkermansia muciniphila*. Secondary measures included: (1) comprehensive metabolic panel (CMP) measures (i.e., fasting levels of blood glucose, calcium, blood urea nitrogen, creatinine, sodium, potassium, bicarbonate, and chloride, serum total protein, serum albumin, bilirubin, ALP, AST, ALT) to assess effect of GP-SPI on blood sugar levels, kidney function, acid/base balance, fluid and electrolyte balance, protein levels, and liver enzymes; and (2) exploratory profiling of fecal microbiome and metaproteome, and targeted metabolomics of bile acids and polyphenol-derived microbial metabolites in serum, feces, and urine to reveal common ecological or functional rules existing in microbiotas of healthy individuals as well as bacterial- or host-derived metabolite biomarkers with diagnostic potential.

The primary outcome for sample size estimates was difference in relative abundance of *Akkermansia muciniphila*. Sample size was estimated with G*Power (F test, ANOVA repeated measures, within factors a priori power analysis) assuming a moderate effect size of f = 0.3, α err prob= 0.05, power = 0.85, number of groups = 1, number of measurements = 4, correlation among repeated measures = 0.5, nonsphericity correction ε = 1, a sample size of 19 participants will be required (actual power 0.86). We aimed to enroll 30 subjects, expecting a 15% attrition rate resulting in a final number of 25 participants completing the study protocol.

An Independent Monitoring Committee (IMC) not directly involved with the study was formed to monitor participant recruitment, retention, adherence, and review safety data (i.e., any adverse events) to assess whether intervention should be continued or suspended.

### LC-MS analysis of polyphenols and bile acids

Serum samples from day 0 and 10 and fecal and urine samples from day –5, 0, 2, 4, and 10 were extracted for polyphenol, phenolic acid, and/or bile acid analysis (*n* = 27 per timepoint).

#### Extraction of polyphenols from serum and urine

To deconjugate polyphenols from their glucuronide and sulfate moieties, 200 µL of serum or urine was digested with 25 µL β-glucuronidase from *Helix pomatia* (Cat # G0876, Sigma-Aldrich, Saint Louis, Missouri, USA) in 200 µL sodium acetate (0.1 M, pH 5). Samples were incubated at 37 °C for 45 min in a water bath. Proteins were precipitated with 2 volumes of ice-cold 100% acetonitrile for 1 h at –20 °C and then centrifuged at 16,000 rcf for 10 min at 4 °C. Supernatant was speed vacuumed overnight at room temperature, while protected from light. Prior to running through UPLC-MS, samples were resuspended in 200 µL 90% acetonitrile, 10% water, and sonicated for 2 min then filtered through Corning® Costar® Spin-X® centrifuge tube filters Nylon membrane, pore size 0.22 μm, for 1.5 min at 13,000 rcf. Flow through was transferred into 300 µL inserts in sampler vials for running through UPLC-MS. Mean percent recovery for internal standard, deuterated hydroxybenzoic acid (d4-HBA), in serum was 68% and for urine was 95%.

#### Extraction of bile acids from serum

Bile acids were extracted from serum (50 µL) using previously described methods^12^. Percent recovery in serum for IS, d4-DCA was 91.47% and for d4-CDCA was 86.81%.

#### Extraction of polyphenols from fecal samples

Fecal samples previously collected in ethanol were mixed with water so they could be frozen and lyophilized. 15 mg of dried fecal sample was extracted twice by incubation at 4 °C with 80% methanol for 1 h on a shaker after vortexing to homogenize sample. After extraction, samples were centrifuged at 15 × 000 *g* for 10 min and the supernatant was collected in a clean microfuge tube. Supernatents were pooled. An aliquot of 80 µL of undigested fecal extract was diluted 10X and used for Folin-Ciocalteu and DMAC assays to detect total polyphenols and PACs, respectively, as previously described^3^. Methanol was evaporated from the remaining pooled supernatant by speed vacuum (CentriVap concentration system with cold trap, Labconco, Kansas City, MO USA). Samples were resuspended in 200 µL sodium acetate (0.1 M, pH 5), and then underwent the same digestion and precipitation steps as with serum described. Supernatants were passed through Oasis Prime HLB 3 cc vac cartridges, 60 mg sorbent (Waters, Item# WT186008056), first with 99.99% water, 0.1% formic acid, then with 70% acetone, 29.9% water, 0.1% formic acid, and then 90% acetonitrile, 9.9% water, 0.1% formic acid after which flow throughs were consolidated and placed in speed vacuum overnight at room temperature in the dark. Prior to running through HPLC-MS, samples were resuspended in 80% methanol in tubes prepared with IS. Resuspended samples were sonicated for 2 min then filtered through Corning® Costar® Spin-X® centrifuge tube filters Nylon membrane, pore size 0.22 μm, for 1.5 min at 13,000 rcf. Flow through was transferred into 300 µL inserts in sampler vials. Lyophilized fecal weights were used for normalization of metabolite concentrations as mg of polyphenols per mg feces. The mean percent recovery of polyphenol IS, d4-HBA, in feces was 104%.

Polyphenols and phenolic acids were detected by UPLC-MS and quantified by external calibration curves using >95% pure standards. While many phenolic acids have previously been identified in urine, plasma, or feces after human GP consumption^43,44^, we quantified 7 phenolic acids (total free metabolite levels after enzymatic deconjugation, as detailed in supplementary methods) known to be gut bacterial metabolites of PACB2 (Supplementary Fig. 3). Polyphenols were quantified by external calibration curves (0, 0.078, 0.156, 0.3125, 1.25, 2.5 µg/mL in 90% acetonitrile) with authentic standards (purity >95%) and corrected using internal standard (IS) calibration (S Table 5). An internal standard (IS) solution containing deuterated hydroxybenzoic acid (Toronto Research Chemicals, catalog #H808452) was used to correct for variability in recovery of polyphenol metabolites. Compounds in samples were separated and analyzed by a UPLC/MS system including the Dionex® UltiMate 3000 RSLC ultra-high pressure liquid chromatography system, consisting of a workstation with ThermoFisher Scientific’s Xcalibur v. 4.0 software package combined with Dionex®’s SII LC control software, solvent rack/degasser SRD-3400, pulseless chromatography pump HPG-3400RS, autosampler WPS-3000RS, column compartment TCC-3000RS, and photodiode array detector DAD-3000RS. After the photodiode array detector the eluent flow was guided to a Q Exactive Plus Orbitrap high-resolution high-mass-accuracy mass spectrometer (MS). Mass detection was full MS scan with low energy collision induced dissociation (CID) from 100 to 1000 m/z in negative ionization mode with electrospray (ESI) interface. Sheath gas flow rate was 30 arbitrary units, auxiliary gas flow rate was 7, and sweep gas flow rate was 1. The spray voltage was –3500 volts with a capillary temperature of 275 °C. The mass resolution was 70,000, or higher. Substances were separated on a PhenomenexTM Kinetex C8 reverse phase column, size 100 × 2 mm, particle size 2.6 mm, pore size 100 Å. The mobile phase consisted of 2 components: Solvent A (0.5% ACS grade acetic acid in LCMS grade water, pH 3–3.5), and Solvent B (100% Acetonitrile, LCMS grade). The mobile phase flow was 0.20 ml/min, and a gradient mode was used for all analyses. The initial conditions of the gradient were 95% A and 5% B; for 30 min the proportion reaches 5% A and 95% B which was kept for the next 8 min, and during the following 4 min the ratio was brought to initial conditions. An 8 min equilibration interval was included between subsequent injections. The average pump pressure using these parameters was typically around 3900 psi for the initial conditions. Putative formulas of polyphenols were determined by performing isotope abundance analysis on the high-resolution mass spectral data with Xcalibur v. 4.0 software and reporting the best fitting empirical formula. Database searches were performed using www.reaxys.com (Elsevier Life Sciences IP Limited) and SciFinder (American Chemical Society).

A panel of 27 bile acids were quantified. Stock solutions (0.5 mg/mL–1 mg/mL) of individual BAs (>95% pure standards) were prepared and stored at –20 °C. Conjugated BAs were dissolved and stored in 50% methanol while unconjugated BAs were in 100% methanol for stability. An internal standard (IS) solution (1 μg/mL each) containing a mixture of CDCA-d4 and DCA-d4, was prepared in 50% methanol. Deuterated BA were used as ISs to correct for variability in recovery of BA species. Using stock solutions, 27 BAs were diluted and pooled such that each was present at a concentration of 5 µg/mL (80% methanol) in a final BA mixture. The BA mixture was further diluted with 80% methanol to give final concentrations of 0.00125 μg/mL, 0.0025 μg/mL, 0.0125 μg/mL, 0.125 μg/mL, 1.25 μg/mL, 2.5 μg/mL, 5 μg/mL (7-point standard curve). For preparation of the calibration curves, 200 μL of IS solution (1 µg/mL) was placed into microfuge tubes and dried using a CentriVap concentrator (LABCONCO) coupled with a CentriVap Cold Trap (LABCONCO). Subsequently, 200 μL of each concentration of BA mix was added to the tube containing the dried ISs prior to injection. Bile acids were analyzed on a Water’s Alliance e2695 HPLC system coupled to a Water’s Acquity QDA mass spectrometer equipped with an electrospray interphase (ESI). Analytes were separated in a Cortecs C18+ column (4.6 × 150 mm, 2.7 μm; Waters Milford, MA, USA) and gradient elution with 0.1% formic acid in water (solvent A) and 0.1% formic acid in acetonitrile (solvent B) at flow rate of 1 mL/min as follows: 0–30 min linear gradient from 65 to 35% B; 30–31 min isocratic at 50% B; 31–31.10 step gradient to 35% B and 31.10–40 min isocratic at 35% B before returning to initial conditions at 60 min for the next analysis. The temperature of the column was maintained at 40 °C and the injection volume was 10 μL. Analytes were detected in full scan mode (ESI + /-, scan range 100–1200 m/z) coupled with selective ion recordings (SIRs). Limit of detection (LOD), limit of quantification (LOQ), and the coefficients of variation (C.V.) for bile acids were previously provided^89^, and these measures are provided for the method used for detection of polyphenol analytes in Supplementary Table 23. Runs were organized so that a full set of timepoints from a participant was run consecutively for each sample set.

### Fecal gDNA extraction for microbial analysis

Ethanol was evaporated from fecal samples by speed vacuum (CentriVap^®^ model #7810014 benchtop centrifugal vacuum concentrator connected to –84 °C cold trap model #7460020, Labconco Corporation, Kansas City, MO, USA). gDNA was extracted from 20 to 90 mg of dried fecal sample using the DNeasy PowerSoil Kit (cat # 47014 QIAGEN, Venlo, Germany) with 0.1 mm glass beads (cat # 13118-400, QIAGEN) according to manufacturer’s instructions. gDNA was eluted with RNase free water and stored at –80 °C.

Fecal gDNA extracted from samples collected at day –5, 0, 2, 4 and 10 (*n* = 27 per timepoint) were provided to Azenta Life Sciences (South Plainfield, NJ) for metagenomic sequencing using the Illumina HiSeq® series platform, with maximum read length of 2 × 150 bp. Adapters were trimmed, and low-quality bases (leading: 6; trailing: 6, sliding window: 4:20) and reads less than 60 bp in length were removed via Trimmomatic^90^. Reads that could be aligned to the human genome (H. sapiens, UCSC hg19) were removed (aligned with Bowtie2^91^ using -reorder -no-hd -no-contain -dovetail). After quality control 91.95% ± 3.76% reads remained. Each sample was de novo assembled using Megahit^92^ (-min-contig-len 500, -presets meta-large). The assembled contigs in each sample were binned using metabat2^93^ and maxbin2^94^. Bins were further refined with Metawrap^95^. The quality of each bin was assessed with CheckM^96^ based on single copy marker genes. Bins with >95% completeness, and <5% contamination and 0% strain heterogeneity were retained as high-quality draft genomes. To improve the analysis, we also downloaded genomes from the HGG constructed by Forster et al.^49^. The assembled high-quality draft genomes and the HGG genomes were dereplicated by using dRep^97^ with 99% average nucleotide identity to obtain nonredundant genomes for further analysis (if the dRep cluster contained the draft genomes from our dataset, we used the best genomes within the assembled genomes as the representative genomes of the clusters). DiTASic^98^ was used to calculate the abundance of the genomes in each sample, estimated counts with *p* > 0.05 were removed, and all samples were downsized to 17 million reads. To identify guilds, correlation coefficients between prevalent genomes were calculated based on the abundance of the genomes across all the samples using the method described by Bland and Altman^99^. The correlations were converted to a correlation distance (1-correlation coefficient) and then clustered using the Ward clustering algorithm. From the top of the clustering tree, permutational MANOVA (9999 permutations, *P* < 0.001) was used to sequentially determine whether the two clades were significantly different to cluster the prevalent bacteria into guilds^51^. The taxonomic assignment of the genomes was performed using GTDB-Tk^100^. Functional annotation was conducted using Prokka^101^. Kyoto Encyclopedia of Genes (KEGG) Orthologue (KO) IDs were assigned to the predicted protein sequences in each genome by HMMSEARCH against KOfam using KofamKOALA^102^. Genes encoding formate-tetrahydrofolate ligase, propionyl-CoA:succinate-CoA transferase, propionate CoA-transferase, 4Hbt, AtoA, AtoD, Buk, and But were identified as described previously^103^. Reference bacterial protein sequences involved in bacterial BA metabolism were downloaded from KEGG and UniProt databases based on the enzyme list reported by Gu et al.^104^. Reference bacterial protein sequences potentially related to polyphenol metabolism were download from NCBI database by searching key words “bacteria” with “β-glucosidase”, “β-glucuronidase”,“alpha-rhamnosidase”, “Quercetin 2”3-dioxygenase”, “ Catechol 2”3-dioxygenase“, “Phloretin hydrolase“, “Quercetinase“, “Vanillin dehydrogenase “, “NADPH-dependent curcumin/dihydrocurcumin reductase (CurA)“, “Daidzein reductase”, “Dihydrodaidzein reductase”, “Tetrahydrodaidzein reductase” and “Dihydrodaidzein racemase”^90,105^. Prokka predicted protein sequences were aligned to the reference databases using BLASTP.

gDNA from fecal samples collected on days –5, 0, and 10 (*n* = 27 per timepoint) was used for 16S rRNA amplicon sequencing. gDNA sample concentrations were normalized to 20 ng/µL and sequenced by Azenta Life Sciences (South Plainfield, NJ). Sequences were analyzed using QIIME (Quantitative Insights Into Microbial Ecology) v2.0.1^106^. Paired-end sequences were imported into QIIME2, demultiplexed, and quality filtered using the q2‐demux plugin. Demultiplexed sequences were denoised, decluttered, and merged with DADA2 using q2-dada2 to generate amplicon sequence variants (ASVs)^107^. Before alignment, twenty base pairs (bps) were trimmed from the beginning of forward and reverse sequences. Based on quality scores, forward reads were not truncated, reverse reads were truncated at 248 bp. Unique features (ASVs) were aligned with mafft^108^ (via q2‐alignment) and used to construct a phylogeny with fasttree2 (via q2‐phylogeny)^109^. Sequences were rarified to 33,296, to not exclude samples. Taxonomy was assigned to ASVs using the q2‐feature‐classifier^110^ and classify‐sklearn Naive Bayes taxonomy classifier trained on the silva_132 (99% OTU identity) classifier^111–113^. The non-rarefied ASV feature table was aggregated at phylum and genus levels to discover features altered by SPI- and GP-SPI-supplementation. Each participant received a copy of their personal 16S rRNA amplicon sequencing results. qPCR was performed to estimate total bacteria and the relative abundance of fecal *A. muciniphila* using previously described methods^6^.

### Fecal metaproteome analyses

Fecal samples from day –5, 0, 2, 4, and 10 (*n* = 27 per timepoint) were used for metaproteomics. Stool sample collection and pre-processing optimized for bacterial protein extraction were performed as described previously^114^. After bacterial protein extraction, proteins were then pelleted and washed with acetone prior to resuspension in 6 M urea and 50 mM ammonium bicarbonate buffer for in-solution trypsin digestion. Desalted and dried tryptic peptides were then labeled with TMT11, with pooled sample as channel 1 for reference. All channels are combined and subjected to MS analysis for identification and quantification.

Dionex ultimate RS3000 joined to Exploris 480 mass spectrometer (ThermoFisher Scientific, San Jose, CA) was operated with a nano-electrospray interface operated in positive ion mode. The solvent system consists of buffer A of 0.1% formic acid in water, and buffer B of 0.1% FA in 80% acetonitrile. Reconstituted peptides were loaded on a 75 μm I.D. × 150 mm fused silica analytical column packed in-house with 3μm ReproSil-Pur C18 beads (100 Å; Dr. Maisch GmbH, Ammerbuch, Germany). The flow rate was set to 300 nL/min, and the gradient was set as 5–35% buffer B in 105 min, followed by 5 min from 35 to 80%, 5 min of 80%, and 5 min of re-equilibration. The spray voltage was set to 2.2 kV and the temperature of the heated capillary was 300 °C. One full MS scan from 350 to 1200 m/z was followed by a data-dependent MS/MS scan of the 15 most intense ions with a dynamic exclusion repeat count of 1 in 20 s. The mass resolution is 60,000 for ms1 and 15,000 for ms2. A real-time internal calibration by the lock mass of background ion 445.120025 was used. All data were recorded with Xcalibur software (ThermoFisher Scientific, San Jose, CA). The peak lists of the raw files were processed and analyzed by Metalab MAG version (The fasta database was the 2.0, now updated to 2.02, https://www.ebi.ac.uk/metagenomics/genome-catalogues/human-gut-v2-0-2). Cysteine carbamidomethylation was selected as a fixed modification; the methionine oxidation, and protein N-terminal acetylation were set for variable modification. Enzyme specificity was set to trypsin, not allowing for cleavage N-terminal to proline. TMTpro 11 was set for MS2 level quantification. Other parameters were used as default. All taxon and function annotations are extracted from corresponding annotations for each genome.

Proteins with assigned clusters of orthologous group (**COG**) categories were used to gather information about affected processes. If a protein had multiple COG categories, its abundance was evenly divided into each category. KEGG was used for functional analysis of identified microbial proteins^115^. KO was used for mapping pathways using the KO Database (genome.jp)^116,117^. The KO system is the basis for cross-species annotation in KEGG, which assigns the KO identifier called the K number representing a functional ortholog that corresponds to a KEGG pathway. Filtering and selection process for protein analyses is described in Supplementary Fig 8.

R/shiny package was used to perform functional enrichment analysis of microbial proteins, using Microbiome Profiler^118^. Human protein-protein interactions were explored using the web resource Search Tool for the Retrieval of Interacting Genes/Proteins (STRING) v.11.5^119^. Active interaction sources used were neighborhood, co-expression, experiments, gene fusions, gene co-occurrence, databases, and text mining, using a medium confidence value (0.400) and medium FDR stringency (5%). Based on Unified Human Gastrointestinal protein (UHGP) catalog^120^, we linked the function annotation between metagenomic predicted proteins and metaproteomics via sequence alignment.

### Statistical analysis

Analyses were conducted and graphed using Prism 8.0.2 (GraphPad Software, La Jolla, California, USA) or with R project (version 3.6.1). One-way ANOVA followed by the Benjamini Hochberg post hoc test was used to screen multi-omics data for a significant effect of SPI or GP-SPI (q ≤ 0.25). Friedman test followed by Nemenyi’s post hoc test was used to find guilds and proteins which were significantly affected and changed by SPI (day –5 vs. 0) or GP-SPI-supplementation (Day 0 vs. Day 2, 4, or 10). Bile acids and polyphenols with more than two timepoints were compared using a one-way ANOVA followed by Dunnett’s post-hoc test comparing all timepoints to study day 0. When comparing two timepoints, a paired Wilcoxon *t* test was used for nonparametric data and paired *t* tests was used for data following normal distribution (as determined by Shapiro-Wilk’s test). The structure of the gut microbial community was displayed as principal coordinate analysis (PCoA) plots based on Bray-Curtis dissimilarity metrics while protein and metabolite profiles were displayed as PCoA plots based on Euclidean distance. Euclidean distance was calculated on the z-score transformed abundance of the proteins. Covariate adjusted principal coordinate analysis (aPCoA)^121^ was used to adjust for inter-subject variation by using subject ID as a factor. Subject stratified PERMANOVA tests were performed on Bray-Curtis dissimilarity metric or Euclidean distance to determine differences across timepoints and between male and female subjects. Correlation analysis between metabolites and guilds was determined by Maaslin2, using the individual as random effect, arcsine square-root transformation (AST) transformed guild abundance.

## Supplementary information

Supplementary information

Supplementary information

## Data Availability

The clinical study record is available under Clinical Trial Registry no. NCT04018066. Metagenomic sequencing data were deposited in Sequence Read Archive under reference PRJNA1157410. Proteomics data were deposited to the ProteomeXchange Consortium via the PRIDE partner repository with the dataset identifier PXD058659.
