# Supplementary material for: Grape polyphenols reduce fasting glucose and increase hyocholic acid in healthy humans: a meta-omics study
Source: NPJ Sci Food. 2025 May 27;9:87. doi: 10.1038/s41538-025-00443-6 (PMC12116990; doi:10.1038/s41538-025-00443-6)
Supplement: Supplementary file 2 — Supplementary information [file 41538_2025_443_MOESM2_ESM.pdf]

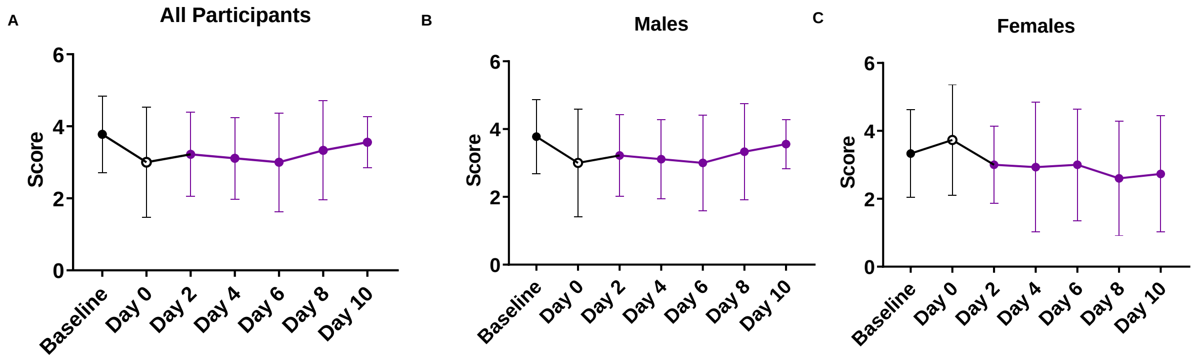

Supplemental Figure 1. Bristol Stool Results. Mean ± SD scores of **(A)** all participants, **(B)** males, and **(C)** females at baseline, after 5 days of SPI-supplementation (Day 0) and after 2, 4, 6, 8 and 10 days of GP-SPI supplementation. Data were analyzed by two-way ANOVA with time and subject as variables, followed by Dunnett's multiple comparison test using day 0 as a control. Significance differences were attributed to subject variability ($F (14,84) = 3.026$, $p = 0.0008$), but not study day ($F (6,84) = 1.108$, $p = 0.3649$).

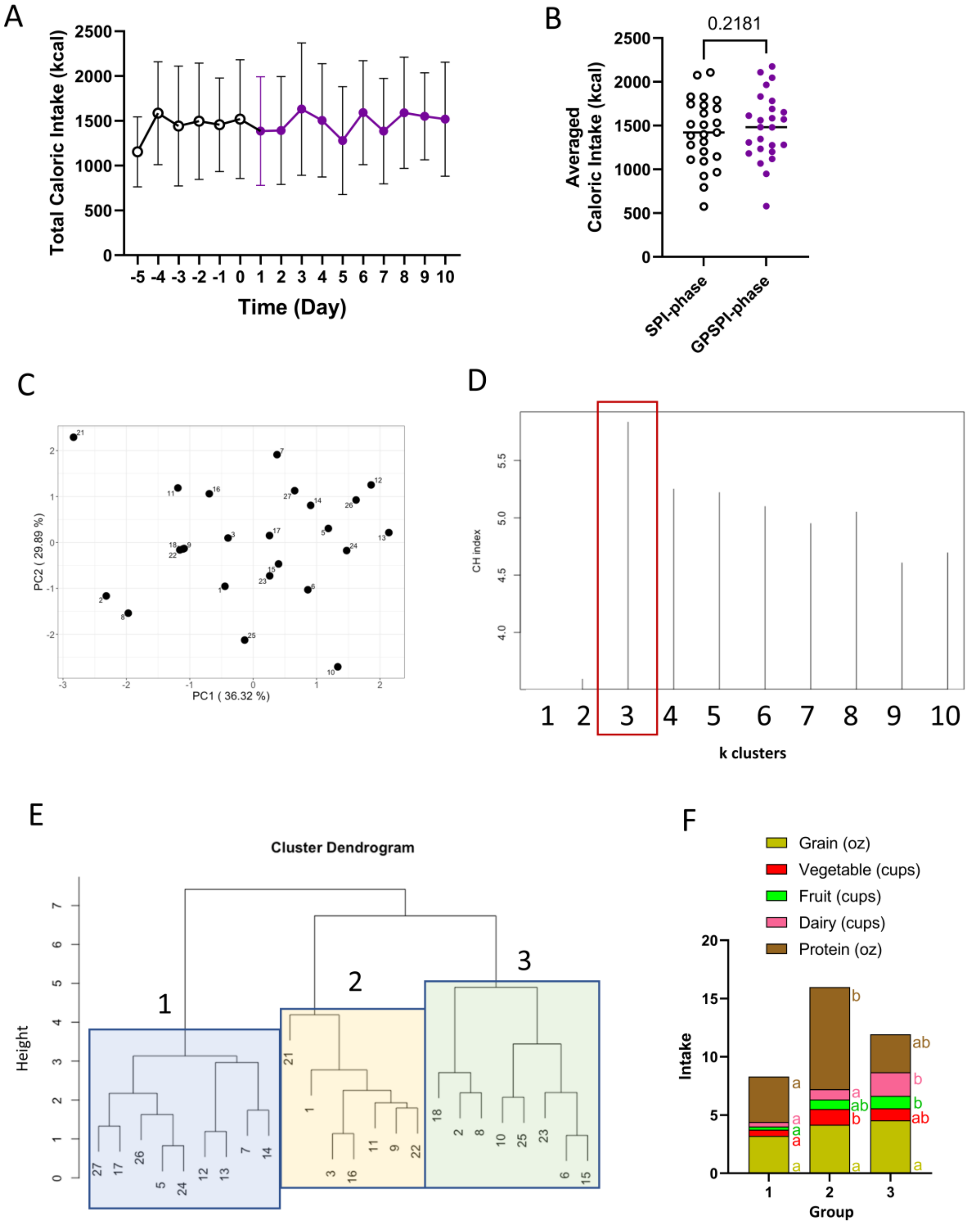

Supplemental Figure 2. Participant food intake patterns. (A) Total caloric intake (mean ± SD) over the study (n = 24; 3 subjects had incomplete datasets for food intake and could not be included in analysis). Significant two-way ANOVA results were obtained for differences in caloric intake by participant, ($F(24, 359) = 9.231$, $p<0.0001$), but not by day, ($F(15, 359) = 1.665$, $p = 0.0559$). **(B)** Average caloric intake during SPI supplementation (Day -5 to Day -1) vs. period of GP-SPI supplementation (Day 1 to Day 10) was not different as determined by paired t-test. **(C)** PCoA based on Euclidean distance between subject intake of grains, vegetables, fruits, dairy, and protein over days 1-16. **(D)** Optimal number of clustering showing the value of 3 with the highest CH index. **(E)** Cluster dendrogram based on Ward linkage showing groups participants were assigned to based on similarity in their food intake. Subject 4 intake had much higher total vegetable and fruit intake than the others and was not assigned to groups 1-3. **(F)** Bar-plot showing distribution between intake of food groups across groups 1-3. Statistical significance between intake of foods between groups 1-3 was determined by Kruskal Wallis test followed by Nemenyi's post hoc test. Different letters denote statistically significant intake of that food group compared to other groups.

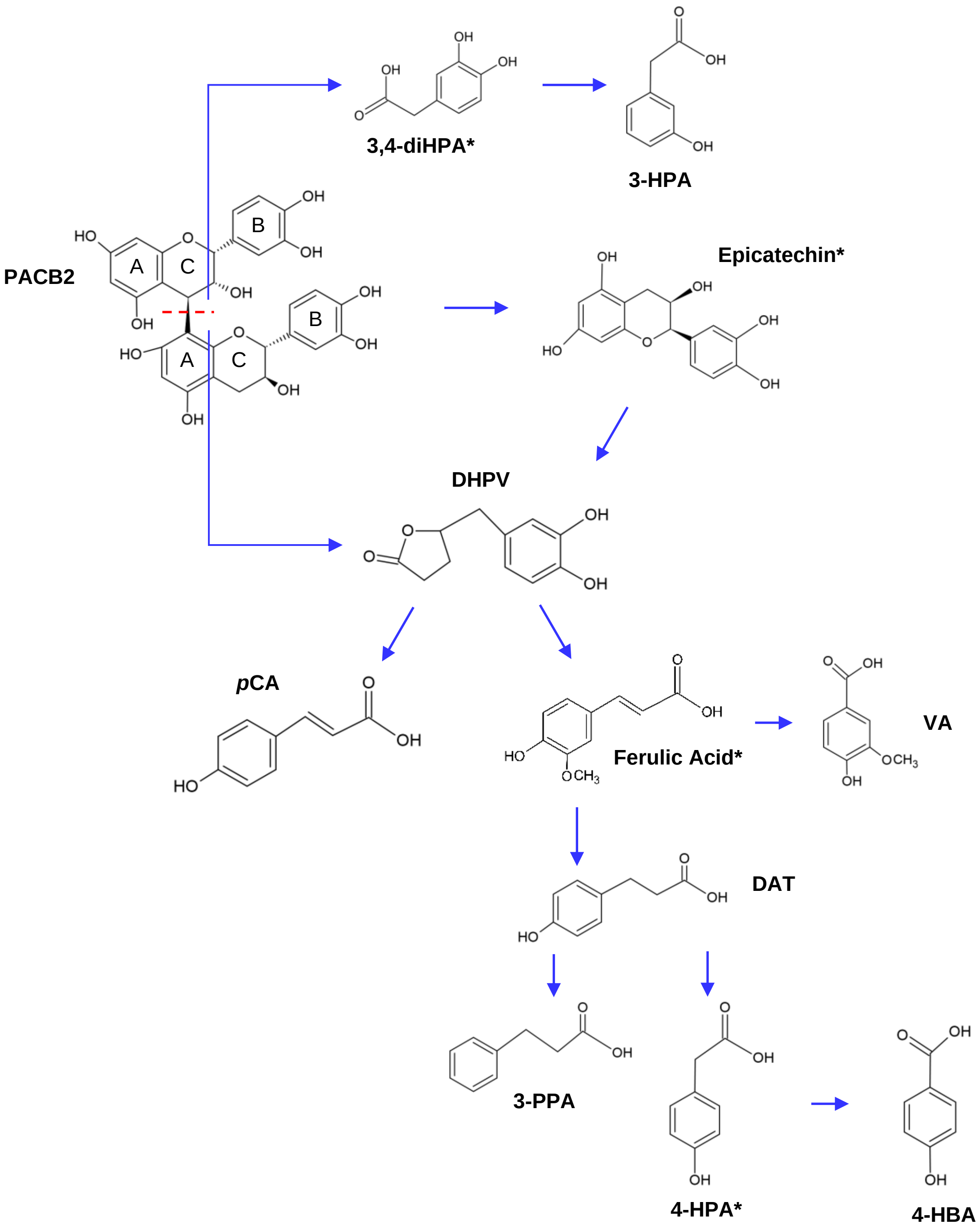

Supplemental Figure 3. Previously reported gut bacterial metabolites of PACB2. Gut bacteria can directly metabolize PACB2 dimer, by cleavage of: 1) the C ring of the upper unit to yield 2-(3,4-dihydroxyphenyl)acetic acid (3,4-diHPA) and 3-hydroxyphenylacetic acid (3-HPA) or 2) the A ring of the lower unit to yield 5-(3',4'-dihydroxyphenyl)-γ-valerolactone (-)-epicatechin (DHPV), para-coumaric acid (*p*CA), ferulic acid, vanillic acid (VA), desaminotyrosine (DAT), 3-phenylpropionic acid (3-PPA) also called hydrocinnamic acid, 2-(4-hydroxyphenyl)acetic acid (4-HPA), and 4-hydroxybenzoic acid (4-HBA). Arrows indicate tentative degradation pathways that may occur via one or more steps. Bacterial cleavage of the interflavan bond of PAC dimers into monomeric flavan-3-ols (i.e., epicatechin) has also been reported (red dashed line), but this reaction accounted for no more than 10% of the metabolized PACB2 and its occurrence depends on individualized capabilities of the gut microbiota. * Epicatechin, 3,4-diHPA, ferulic acid and 4-HPA are shown to illustrate reported conversions but were not measured in our study. Structures were drawn in ChemSketch version 2024.

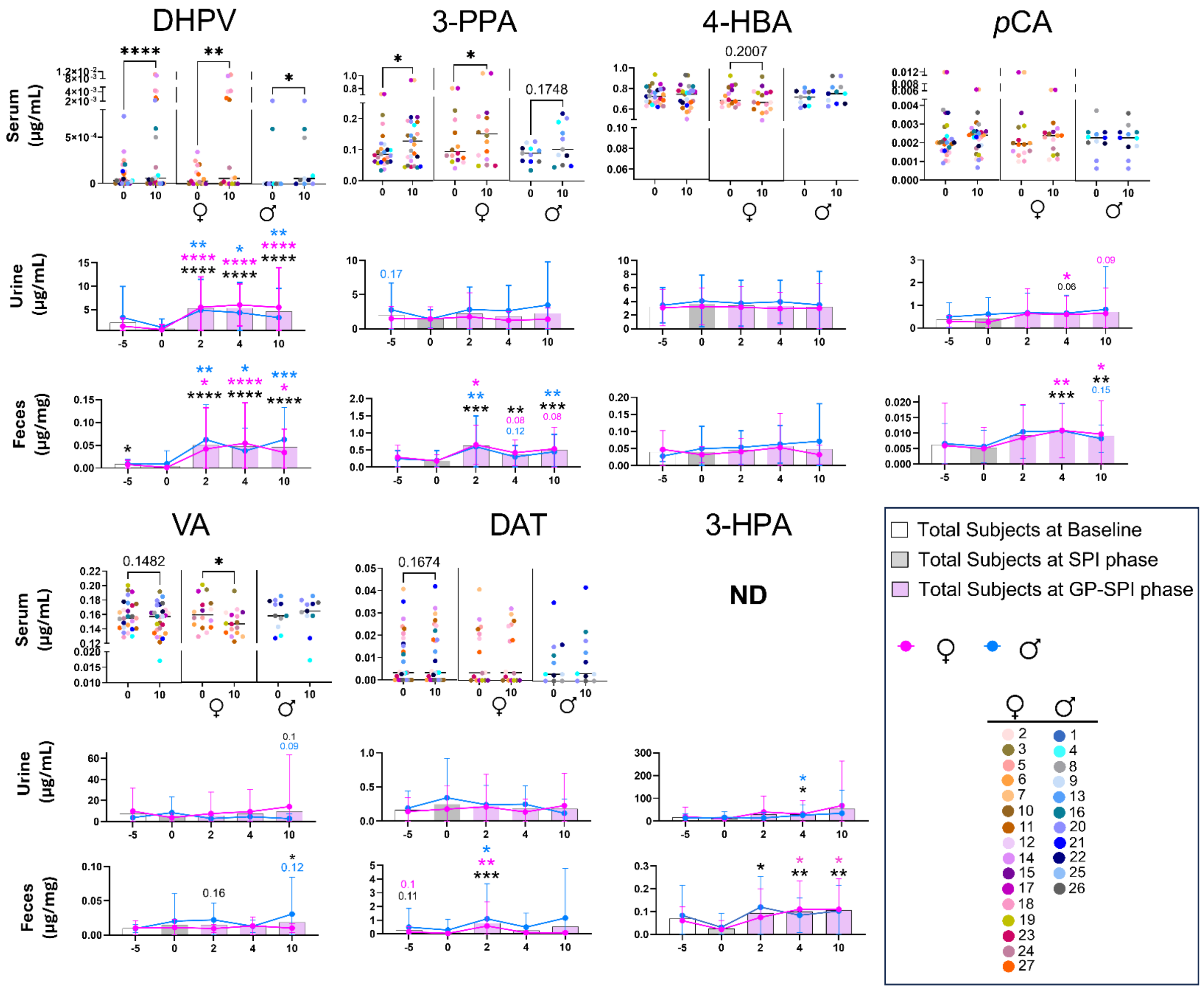

Supplemental Figure 4. PAC-derived microbial metabolites detected in serum, urine, and feces. Values are presented as scatter plots for serum (with each point representing a participant), and as mean ± SD for urine and feces. At indicated time points, bar graph portion represents all subjects while pink line graph represents females, and blue line graph represents males. 3-HPA was not detected (ND) in serum. Based on outcome of Shapiro-Wilk's test, difference between day 0 and day 10 serum samples were determined by nonparametric, paired Wilcoxon test. For urine and feces, difference was determined via Freidman test followed by Dunnet's post hoc test comparing all timepoints to day 0. *p< 0.05; **p< 0.001; ***p< 0.0001.

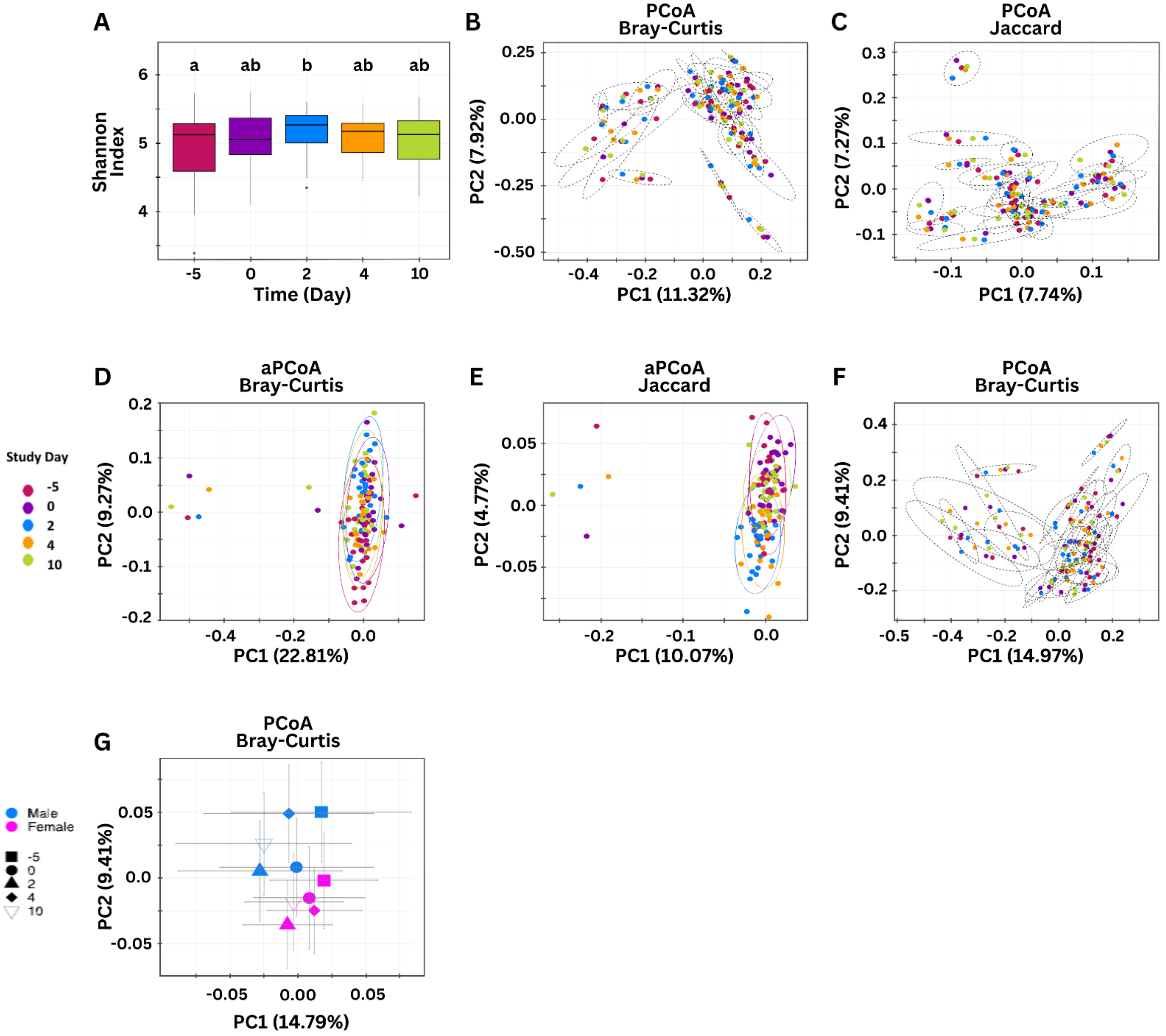

Supplemental Figure 5. Changes to microbial genomes and guilds induced by SPI and GP-SPI. (A) Alpha-diversity metric, Shannon index. Differences were determined by Freidmans test followed by Nemenyi's post hoc test. Different letters denote statistical significance, $p<0.05$. Principal Component Analysis (PCoA) of **(B)** Bray-Curtis, and **(C)** Jaccard distance between genomes. adjusted PCoA (aPCoA) of **(D)** Bray-Curtis, and **(E)** Jaccard distance between genomes. PCoA of Bray-curtis between guilds of **(F)** all subjects, and **(G)** mean of males and females.

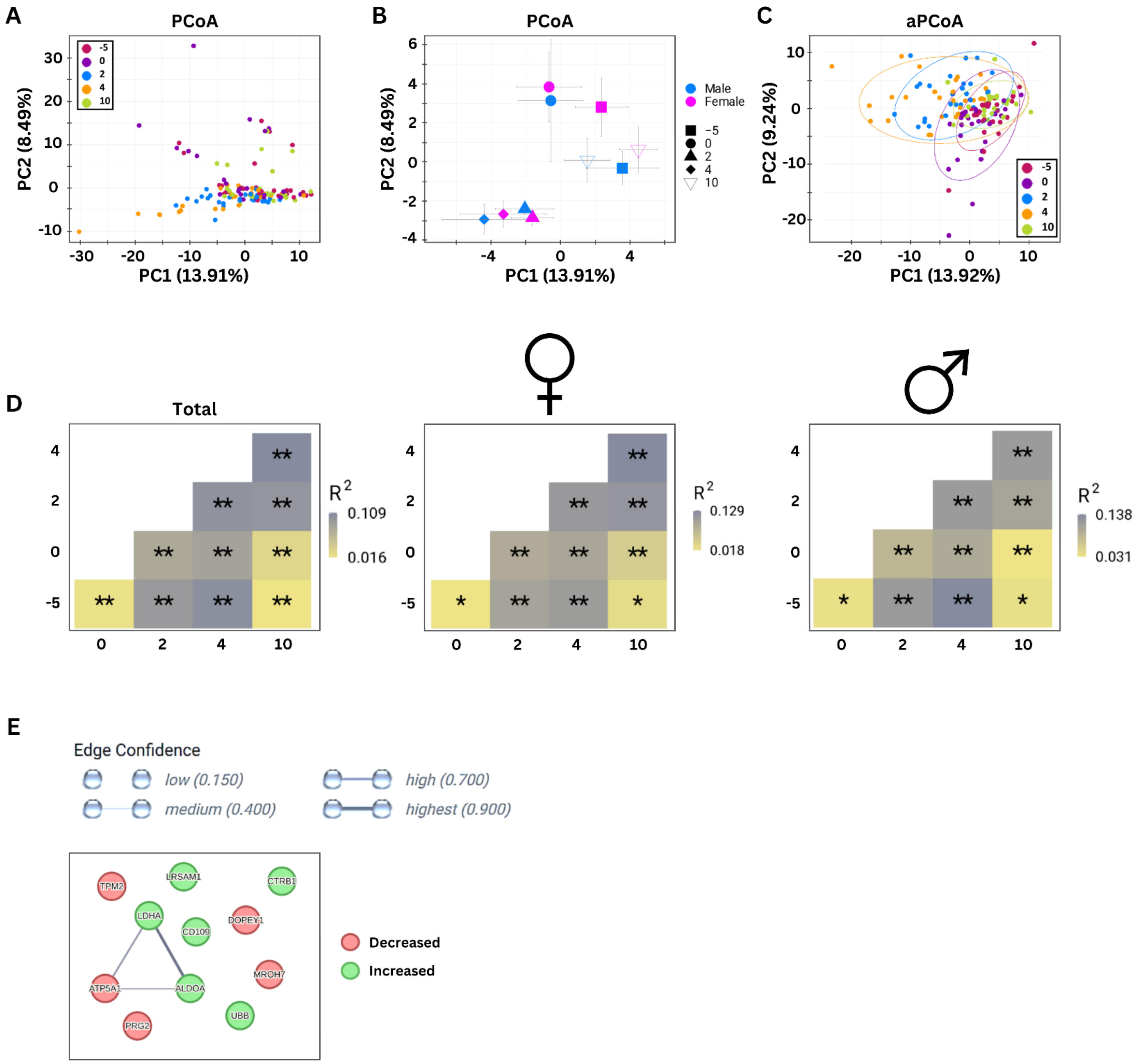

Supplemental Figure 6. GP-SPI induced transient changes to host proteins. PCoA plot based on Euclidean distance showing **(A)** individual subjects, and **(B)** mean ± SD of males vs females at each timepoint. **(C)** aPCoA plot based on Euclidean distance, dots represents all subjects at the different timepoints. **(D)** Subject stratified PERMANOVA test results based on Euclidean distance of total participant pool, males, or females. **(E)** Network of proteins decreased (red) or increased (green) over 2 or more consecutive timepoints during the GP-SPI-phase per Nemenyi post hoc test (by a threshold of q≤ 0.25), showing predicted or known interactions based on String version 11.5.

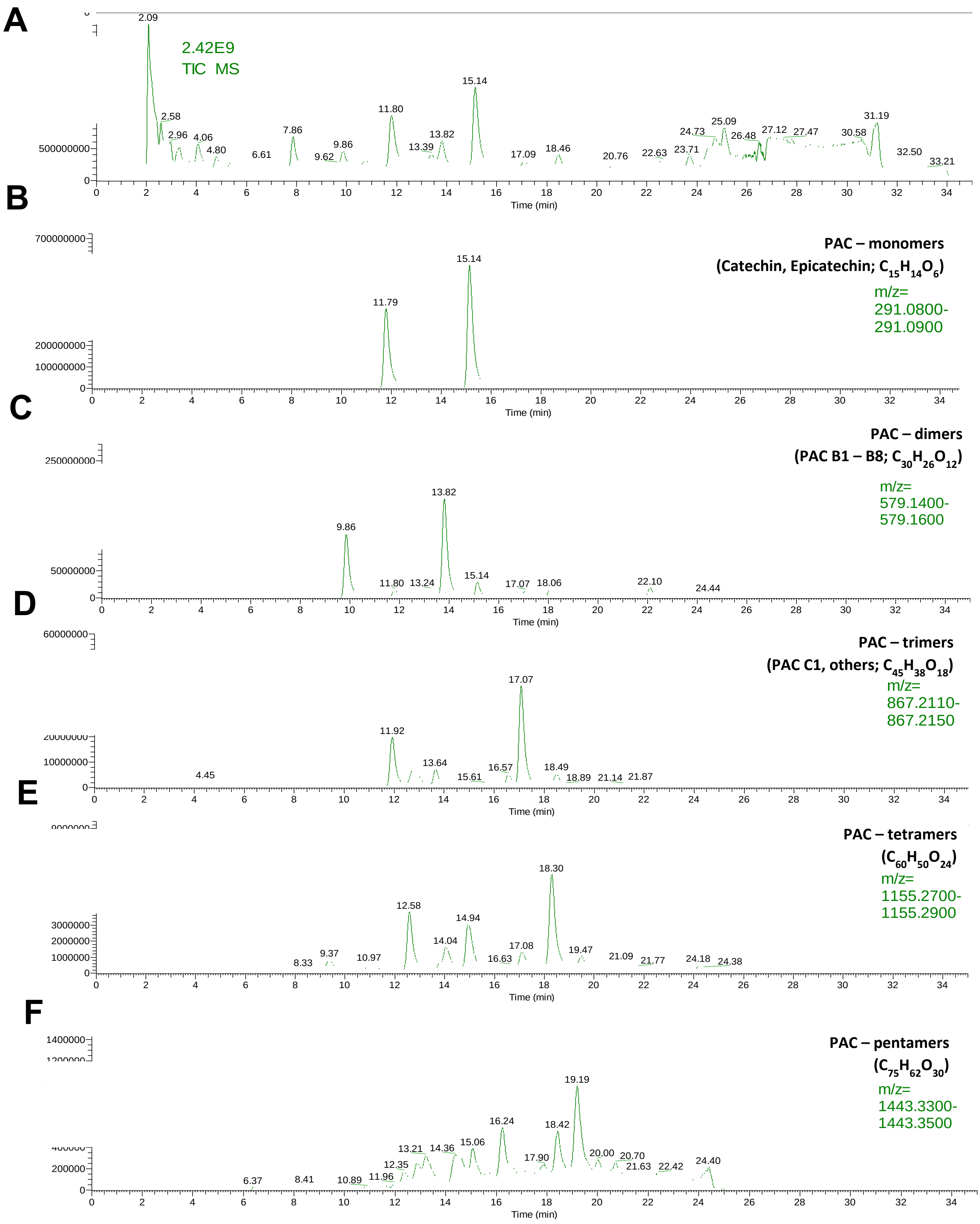

A
2.42E9
TIC MS
2.09
2.58
2.96
4.06
4.80
6.61
7.86
9.62
9.86
11.80
13.39
13.82
15.14
17.09
18.46
20.76
22.63
23.71
24.73
25.09
26.48
27.12
27.47
30.58
31.19
32.50
33.21
500000000
Time (min)
B
PAC – monomers
(Catechin, Epicatechin; $C_{15}H_{14}O_6$)
m/z=
291.0800-
291.0900
700000000
200000000
100000000
11.79
15.14
Time (min)
C
PAC – dimers
(PAC B1 – B8; $C_{30}H_{26}O_{12}$)
m/z=
579.1400-
579.1600
250000000
50000000
9.86
11.80
13.24
13.82
15.14
17.07
18.06
22.10
24.44
Time (min)
D
PAC – trimers
(PAC C1, others; $C_{45}H_{38}O_{18}$)
m/z=
867.2110-
867.2150
60000000
10000000
4.45
11.92
13.64
15.61
16.57
17.07
18.49
18.89
21.14
21.87
Time (min)
E
PAC – tetramers
($C_{60}H_{50}O_{24}$)
m/z=
1155.2700-
1155.2900
3000000
2000000
1000000
8.33
9.37
10.97
12.58
14.04
14.94
16.63
17.08
18.30
19.47
21.09
21.77
24.18
24.38
Time (min)
F
PAC – pentamers
($C_{75}H_{62}O_{30}$)
m/z=
1443.3300-
1443.3500
1400000
200000
6.37
8.41
10.89
11.96
12.35
13.21
14.36
15.06
16.24
17.90
18.42
19.19
20.00
20.70
21.63
22.42
24.40
Time (min)

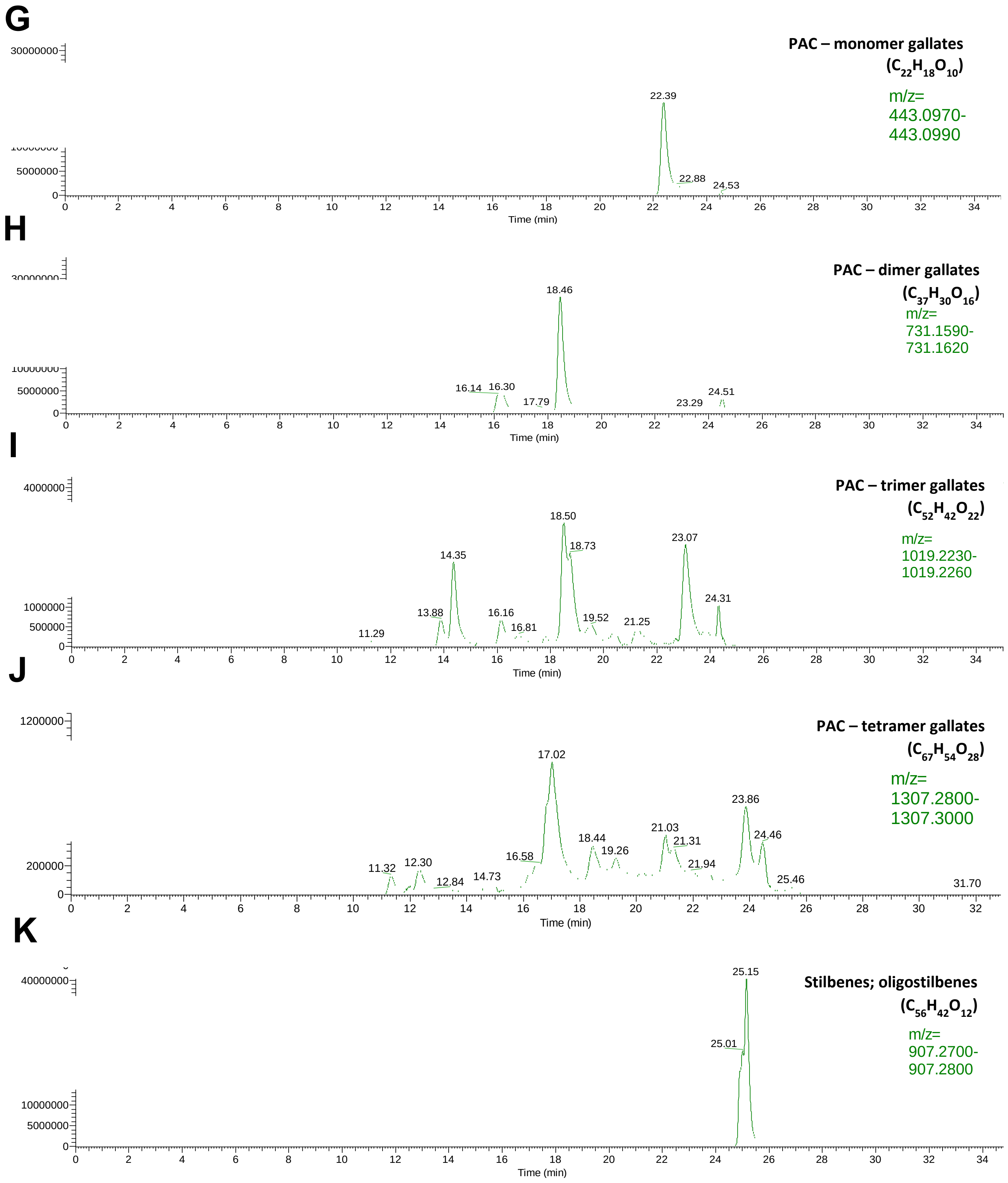

Supplemental Figure 7. Putative polyphenol species with best fitting empirical formulas and mass-to-charge (m/z) ratio, detected in GP-extract by UPLC-MS and Xcalibur v. 4.0 software. Chromatograms of **(A)** all compounds, and identified PAC **(B)** monomers, i.e., catechin and epicatechin, **(C)** dimers (e.g., PAC B1 – B8), **(D)** trimers, i.e., PAC C1, **(E)** tetramers, **(F)** pentamers, **(G-J)** PAC monomer, dimer, trimer, tetramer gallates, and **(K)** stilbenes and oligostilbenes.

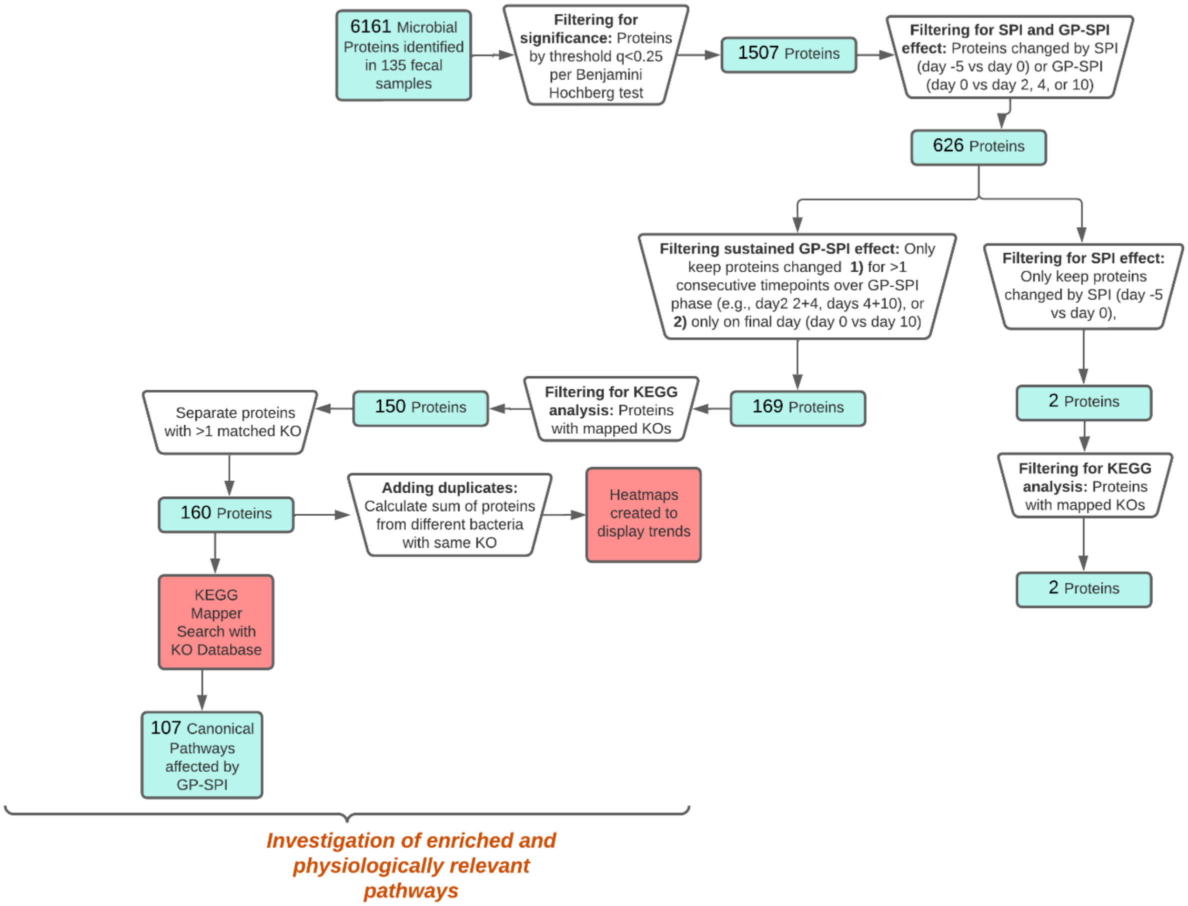

Supplemental Figure 8. Diagram outlining screening process for microbial proteins affected by SPI and GP-SPI and functional analyses. Created with Lucid.
